# A genome-wide association study of freezing tolerance in red clover (*Trifolium pratense* L.) germplasm of European origin

**DOI:** 10.3389/fpls.2023.1189662

**Published:** 2023-05-10

**Authors:** Stefano Zanotto, Tom Ruttink, Marie Pégard, Leif Skøt, Christoph Grieder, Roland Kölliker, Åshild Ergon

**Affiliations:** ^1^ Faculty of Biosciences, Norwegian University of Life Sciences (NMBU), Ås, Norway; ^2^ Plant Sciences Unit, Flanders Research Institute for Agriculture, Fisheries and Food (ILVO), Melle, Belgium; ^3^ INRAE P3F, Lusignan, France; ^4^ IBERS, Aberystwyth University, Aberystwyth, United Kingdom; ^5^ Group of Fodder Plant Breeding, Agroscope, Zurich, Switzerland; ^6^ Molecular Plant Breeding, Institute of Agricultural Sciences, ETH Zurich, Zurich, Switzerland

**Keywords:** cold stress, forage legumes, GWAS, haplotypes, linkage disequilibrium, LT50, pool-GBS, winter survival

## Abstract

Improvement of persistency is an important breeding goal in red clover (*Trifolium pratense* L.). In areas with cold winters, lack of persistency is often due to poor winter survival, of which low freezing tolerance (FT) is an important component. We conducted a genome wide association study (GWAS) to identify loci associated with freezing tolerance in a collection of 393 red clover accessions, mostly of European origin, and performed analyses of linkage disequilibrium and inbreeding. Accessions were genotyped as pools of individuals using genotyping-by-sequencing (pool-GBS), generating both single nucleotide polymorphism (SNP) and haplotype allele frequency data at accession level. Linkage disequilibrium was determined as a squared partial correlation between the allele frequencies of pairs of SNPs and found to decay at extremely short distances (< 1 kb). The level of inbreeding, inferred from the diagonal elements of a genomic relationship matrix, varied considerably between different groups of accessions, with the strongest inbreeding found among ecotypes from Iberia and Great Britain, and the least found among landraces. Considerable variation in FT was found, with LT50-values (temperature at which 50% of the plants are killed) ranging from -6.0°C to -11.5°C. SNP and haplotype-based GWAS identified eight and six loci significantly associated with FT (of which only one was shared), explaining 30% and 26% of the phenotypic variation, respectively. Ten of the loci were found within or at a short distance (<0.5 kb) from genes possibly involved in mechanisms affecting FT. These include a caffeoyl shikimate esterase, an inositol transporter, and other genes involved in signaling, transport, lignin synthesis and amino acid or carbohydrate metabolism. This study paves the way for a better understanding of the genetic control of FT and for the development of molecular tools for the improvement of this trait in red clover through genomics assisted breeding.

## 1 Introduction

Red clover (*Trifolium pratense* L.) is the most important perennial forage legume in Northern Europe (Helgadóttir et al., 2014; Annicchiarico et al., 2015), where it is mainly cultivated in mixture with grasses under a cutting regime for the production of silage. Its capability to fix atmospheric nitrogen through symbiotic nitrogen fixation significantly reduces the requirement for nitrogen fertilization (Jensen et al., 2012; Lüscher et al., 2014; Reckling et al., 2016). Thanks to this, and to the high protein content (Frame et al., 1997), red clover plays an important role in the shift towards a more sustainable agriculture in Europe, which is currently not self-sufficient when it comes to the production of protein for food and feed (de Visser et al., 2014; Voisin et al., 2014). However, the cultivation of red clover at northern latitudes is hampered by its poor field persistency, mainly caused by low winter survival (Abberton and Marshall, 2005). The improvement of persistency is one of the main breeding goals for the species and will result in both higher forage yield and protein content of mixed red clover - grass swards (Marshall et al., 2017).

Freezing temperatures are one of several causes of winter mortality at locations with continental climate characterized by harsh winters (Bélanger et al., 2006). The ability to tolerate low freezing temperatures (freezing tolerance, FT) is largely dependent on a period of cold acclimation (CA) at low above-zero temperatures. FT measured under controlled conditions was successfully used as a determinant for freezing tolerance and field winter survival in winter wheat (*Triticum aestivum;* Gusta et al., 2001), perennial ryegrass (*Lolium perenne;* Waldron et al., 1998; Hulke et al., 2008) and white clover (*T. repens;* Annicchiarico et al., 2001). However, a more prolonged test, LD50 (lethal duration time for 50% kill), was proposed as a better proxy for winter survival of winter canola (*Brassica napus*) in the field than LT50 (Waalen et al., 2011). A previous study in red clover of Nordic origin (Zanotto et al., 2021), found that FT (calculated as LT50 with the same method as used here) was significantly correlated with winter survival at a continental location characterized by low freezing temperatures in the winter, showing that the LT50 method reveals relevant variation. However, the same study also identified a considerable genotype-by-environment (G×E) interaction on winter survival, which underlines the complex nature of this trait. FT can be improved by selection and breeding (Bertrand et al., 2016), but little is known about the genetic control of FT in red clover. Previous studies identified regions in the red clover genome associated with persistency under various biotic and abiotic stresses (Herrmann et al., 2008; Klimenko et al., 2010; Ergon et al., 2019), but none of these studies specifically analyzed the level of FT of the plant material.

Red clover is a natural diploid (2n = 2x = 14) outbreeding species with a genome size of approximately 420 Mb (Sato et al., 2005). A draft genome for the species (309 Mb, of which 164 Mb are placed on chromosomes) was published in 2015 by De Vega et al., facilitating genomic studies. Recently, a new chromosome-scale assembly of 413.5 Mb was published (Bickhart et al., 2022). Red clover is highly self-incompatible, has a very polymorphic genome, and usually a higher genetic diversity within than between populations (Jones et al., 2020). Red clover cultivars are commonly bred as synthetic populations with up to twenty or more parents. A recent study revealed that a high amount of genetic diversity is maintained within Nordic red clover cultivars created by breeding programs (Osterman et al., 2021). Furthermore, there is a large amount of genetic variation for both FT and other traits available among landraces, wild populations and old cultivars in the Nordic Genetic Resource Center (NordGen; Zanotto et al., 2021). Variation in such material can be exploited to improve cultivars for specific target traits (Kouamé and Quesenberry, 1993; Greene et al., 2004; Dias et al., 2008; Annicchiarico and Pagnotta, 2012), including traits related to persistency such as winter survival and freezing tolerance (Zanotto et al., 2021).

Genotyping-by-sequencing (GBS) of DNA pools is a time- and cost-effective method for genotyping a large number of accessions, which is useful when genetic characterization is more relevant at population level than at the constituent individual level, such as in plant breeding of self-incompatible forage species (Byrne et al., 2013). Genome-wide association studies (GWAS) have been successfully used in forage legumes to elucidate the genetic control of complex traits (Biazzi et al., 2017; Inostroza et al., 2018). GWAS analysis may be confounded by population structure and variation in relatedness among accessions (Korte and Farlow, 2013). Therefore, it is important to use GWAS models that can account for this in order to reduce the risk of identifying false positive associations (Liu et al., 2016). In GWAS analysis, haplotype data can be expected to complement single nucleotide polymorphism (SNP) data, as haplotypes may reveal associations that are not detectable when using SNP data only (Hamblin and Jannink, 2011; Bekele et al., 2018; Ergon et al., 2022).

The goal of this work was to characterize the level of FT of a diverse panel of red clover accessions and to identify genomic regions and candidate genes associated with FT through a GWAS.

## 2 Materials and methods

### 2.1 Plant material and phenotyping

A total of 393 red clover accessions were phenotyped for FT under controlled experimental conditions. These accessions are cultivars, breeding material, landraces and ecotypes mostly of European origin (**Table 1**; **Supplementary Table 1**) collected and characterized within the EUCLEG project^1^ (Nay et al., 2023). FT was determined in young plants after a short cold acclimation treatment (two weeks at 3–4°C, 12 h photoperiod and 110 μmol m^-2^s^−1^ photosynthetic photon flux density (PPFD) and expressed as LT50 (temperature required to kill 50% of the plants), in a similar way as described by Zanotto et al. (2021). The experiment was organized in eight incomplete blocks, each containing half of the accessions. Accessions were randomized among blocks, so that each accession was represented in four of the eight blocks. Due to space limitation, only one block could be freeze-tested at the time, every two weeks. Sowing, pricking and cold acclimation prior to the freezing test was therefore staggered in a sequential order. Each block consisted of twelve sub-blocks with one plant per accession. Three sub-blocks per accession and block were exposed to one of four different testing temperatures within the range from -5°C, to -17°C (temperatures were adjusted after freeze-testing block 1 and 2 in order to align with the variation in FT). In total, across all blocks, each accession was represented by 48 plants tested at various temperatures. Survival data from the four blocks per accession were pooled before LT50 was estimated using the invest function of the “investr” package in R (Greenwell and Kabban Schubert, 2014).

**Table 1:** The 393 red clover (*Trifolium pratense*) accessions used in this study, distributed across countries or regions and population types.

Region	Cultivars	Breeding material	Landraces	Ecotypes
Americas (Argentina, Canada, USA)	11	5		
Belgium	8	4	4	
Central or eastern Europe (Austria, Bulgaria, Poland, Slovakia)	1			4
Czech Republic	26	21		2
Denmark	3			
Finland	1		3	1
France	11			
Germany	4			
Great Britain	11	5	1	3
Italy				4
Norway	5	18	1	
Oceania/Asia (Japan, New Zealand)	12			
Serbia	10	14		5
Iberia (Portugal, Spain)				9
Sweden	15	58	32	
Switzerland	15	5	61	

### 2.2 Genotyping

The 393 accessions were genotyped at the accession level using pool-GBS (Elshire et al., 2011; Byrne et al., 2013) of DNA extracted using the DNAeasy 96 well kit (QIAGEN) from pools of the first emerging leaf of 200 seedlings per accession. GBS was performed by the LGC Group^2^, using i) a combination of *Pst*I and *Mse*I for digestion, ii) a methodological adaptation that involves molecular normalization of read depth across loci within samples and size selection (range 100-250 bp, peak around 175 bp), and iii) paired-end sequencing. 

SNP calling and allele frequency estimation was done as described in Keep et al. (2020); details regarding the parameters specifically used in the present study are provided in the **Supplementary Material 1**. One accession (EUC_TP_107) had more than 80% missing values across SNPs and was discarded from further analyses, leaving 392 accessions. Only biallelic SNPs with a read depth between 30 and 500 were considered. Also, SNPs with more than 5% missing values or overall minor allele frequency (MAF) < 0.05 were removed. This resulted in a set of 20,156 SNPs of which 12,777 (63%) were located on chromosomes and 7,379 on unanchored scaffolds. The number of missing values per marker and per individual, as well as the allele frequency distribution, after filtering, is shown in **Supplementary Figures 1**, **2**. Missing data (0.72% of all sample and SNP genotype calls) were imputed by replacing each missing data point with the mean allele frequency across all accessions per SNP.

Haplotype variants within GBS loci were called and their relative frequencies estimated with the SMAP package (Schaumont et al., 2022) as explained in Ergon et al. (2022), using the SNP data set achieved when removing only SNPs with more than 20% missing data as input. In the process, haplotype variants with an overall MAF < 0.05 were discarded. We then defined a haplotype polymorphism (HTP) as a GBS locus that contains two or more haplotype variants. We removed HTPs with missing values in more than 5% of the samples. This resulted in a data set with allele frequencies of 20,745 haplotype variants in 7,477 HTPs, giving an average of 2.8 variants per HTP. The number of missing values per haplotype and per accession, as well as the haplotype allele frequency distribution after filtering, are shown in **Supplementary Figures 3**, **4**. The data set was then imputed for missing values (0.38%) as explained above for the SNP data set. Overall, 13,359 haplotype variants (64%) were located on chromosomes and 7,389 (36%) on unanchored scaffolds, within 4,833 and 2,644 HTP loci, respectively. The set of 4,833 HTP loci located on the chromosomes was used to obtain a measure of marker distribution across the genome in our material.

### 2.3 Population structure, genomic relationship and linkage disequilibrium (LD)

The population structure among the 392 accessions was investigated with a principal component analysis (PCA) based on the reference allele frequencies of the SNPs after filtration and imputation. SNP- and haplotype-based genomic relationship matrices (GRM) were calculated following the method described by Ashraf et al. (2016). The method is based on ‘method 1’ in VanRaden (2008) and adapted for the use of allele frequency data. The genotype matrix (**F** *_ij_*, with *i* indexing the samples and *j* indexing the markers) was centered by the mean allele frequencies $\left(\bar{F_{j}}\right)$ , and the resulting genotype matrix $M(M_{ij}=F_{ij}-\bar{F_{j}})$ was used to compute the GRM ***G***, as follows


G=MM'1n∑j=1mp^j(1−p^j)

where *m* represents the number of markers, ${\hat{p}}_{j}$ is the estimated allele frequency of the *j^th^* marker, and *n* represents the total ploidy number, which is the sum of the ploidy number of the parents used to generate the populations as demonstrated by Ashraf et al. (2016). We used *n* = 16, as suggested by Cericola et al. (2018) for synthetic perennial ryegrass populations. GRMs were calculated with higher *n* values, and a negligible effect was found in the GWAS results using these different GRMs (data not shown), therefore a value of *n* = 16 was used throughout this study.

Because we were using population allelic frequencies it was not feasible to assess the true LD between SNP markers in the population. Instead, the LD was estimated as the squared partial correlation between the reference allele frequencies of pairs of SNPs *r* ^2^ *_s_*; (Lin et al., 2012; Mangin et al., 2012). This measure removes the bias on the correlation between reference allele frequencies at different loci that is due to kinship. *r* ^2^ *_s_* values were calculated in R (R Core Team, 2020), using the “pcor.shrink” function of the “corpcor” package (Schafer et al., 2021).

### 2.4 GWAS and candidate gene search

Different SNP-based GWAS models were initially performed using the “rrBLUP” package (Endelman, 2011) with and without correction for kinship (as expressed by the GRM) and/or population structure (as expressed by principal component scores), but none of the models resulted in any significant associations. SNP- and haplotype-based GWAS for FT were therefore performed with the Multi Locus Mixed Model (MLMM), which incorporates multiple markers identified as significantly associated with the phenotypic trait as covariates simultaneously (Segura et al., 2012). We included the GRM in the models in order to account for kinship. The analyses were performed in R with the “mlmm.gwas” package (Bonnafous et al., 2019). The model with the lowest extended Bayesian information criterion (e-BIC) value (Chen and Chen, 2008) was selected as the best model, and SNPs and haplotypes with a *p*-value lower than the Bonferroni threshold were considered as significant. For each of the significant markers (SNPs and haplotypes) a linear regression between the reference allele frequency and FT was calculated to estimate the allele effect on the phenotype (slope) and the phenotypic variance explained (*R* ^2^). Furthermore, to characterize the effect of kinship on the *R* ^2^, two different regression models were fitted, one without and one with kinship (as a random genetic effect), with the “lmekin” and the “lm” functions of the “coxme” (Therneau, 2020) and “stats” (R Core Team, 2020) packages, respectively. The variance explained by regression models including all the significant markers simultaneously was also calculated, again with and without including the kinship matrix. All the *R* ^2^ values were estimated from the analyses output with the function “r.squaredLR” of the package “MuMin” (Barton, 2020). All the statistical analyses were performed in R (R Core Team, 2020).

Given the limited LD found, the nearest gene to significant SNPs and haplotypes identified by the GWAS were considered as candidate genes. They were identified in the red clover genome Tp2.0 (De Vega et al., 2015) with the gbrowse function available within the Legume Information System^3^. Gene-coding sequences (CDS) were used as query in BLASTn searches (blast.ncbi.nlm.nih.gov) in the *Arabidopsis thaliana* and *Medicago truncatula* genome to identify the most similar genes in these model species.

## 3 Results

### 3.1 Variation in freezing tolerance

**Figure 1:**
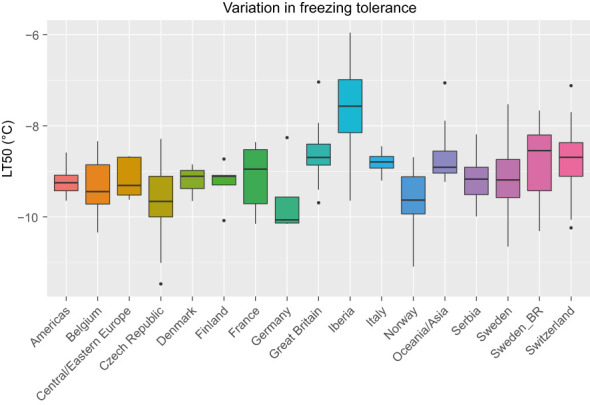
Variation in freezing tolerance (LT50, in °C) among 392 red clover accessions from different countries or regions. F2 breeding populations from Sweden (Sweden_BR) were kept as a separate group. See **Table 1** for number of accessions belonging to each geographical group.

LT50 among the 392 phenotyped accessions ranged between -11.5°C and -6.0°C with an average of -9.1°C. The average standard error among the estimated LT50-values was 0.5°C. The variation in LT50 across different geographical origins is shown in **Figure 1**. The most freezing tolerant accessions were found among the Czech, Norwegian and Swedish material while the most susceptible accessions were found among the Iberian material (note that higher LT50 corresponds to lower FT). Even though only nine accessions were assigned to the Iberian group, this group had the largest variation in FT.

### 3.2 Marker density, LD and genomic relationship among accessions

Since several SNP markers are often clustered within the same GBS locus, the number of HTP loci is more representative of the marker density than the number of SNPs or haplotype variants. The average distance between the 4833 HTP loci that mapped to the 164.2 Mb of the genome assembly assigned to chromosomes was 34 kb (see **Supplementary Figure 5** for an illustration of SNP and HTP density along the chromosomes). The average LD-values (*r* ^2^ *_s_*) between SNP markers was very low (0.005-0.01) at distances ranging from 1 Mb to 1 kb and increased to 0.16 at distances shorter than 0.5 kb (**Table 2**; **Supplementary Figure 6**).

**Table 2:** Linkage disequilibrium (LD) at different distances along the seven red clover chromosomes.

	1Mb	0.5Mb	10kb	5kb	2.5kb	1kb	0.5kb^1^	Mean^2^
Chr1	0.0049 (318)	0.0054 (258)	0.0081 (393)	0.0089 (129)	0.0103 (222)	0.0098 (261)	0.2074 (4446)	0.0048
Chr2	0.0047 (165)	0.0043 (377)	0.0068 (274)	0.0108 (102)	0.0155 (116)	0.0125 (240)	0.1680 (4144)	0.0048
Chr3	0.0043 (333)	0.0049 (251)	0.0070 (351)	0.0077 (287)	0.0087 (310)	0.0171 (278)	0.2018 (4661)	0.0048
Chr4	0.0045 (178)	0.0053 (310)	0.0068 (213)	0.0063 (115)	0.0119 (169)	0.0090 (261)	0.1637 (2810)	0.0050
Chr5	0.0055 (112)	0.0067 (163)	0.0058 (169)	0.0051 (77)	0.0062 (100)	0.0081 (334)	0.1089 (2455)	0.0063
Chr6	0.0048 (166)	0.0054 (113)	0.0061 (283)	0.0073 (154)	0.0100 (142)	0.0103 (249)	0.1385 (2407)	0.0052
Chr7	0.0048 (117)	0.0052 (185)	0.0081 (212)	0.0071 (205)	0.0078 (165)	0.0092 (310)	0.1662 (3161)	0.0049
Mean^3^	0.0048	0.0053	0.0070	0.0076	0.0101	0.0109	0.1649	0.0048

LD was calculated according to Lin et al. (2012) and Mangin et al. (2012), as the average squared partial correlations (*r* ^2^ *_s_*) between allele frequencies of SNP pairs at given distances ± 0.5 kb. The number of pairs for each chromosome and distance is given in parentheses. ^1^Average LD in the interval 0-0.5 kb distance between pairs of SNP markers. ^2^ Mean LD across the selected distances for each chromosome.^3^ Mean LD across all chromosomes for each selected distance.

**Figure 2:**
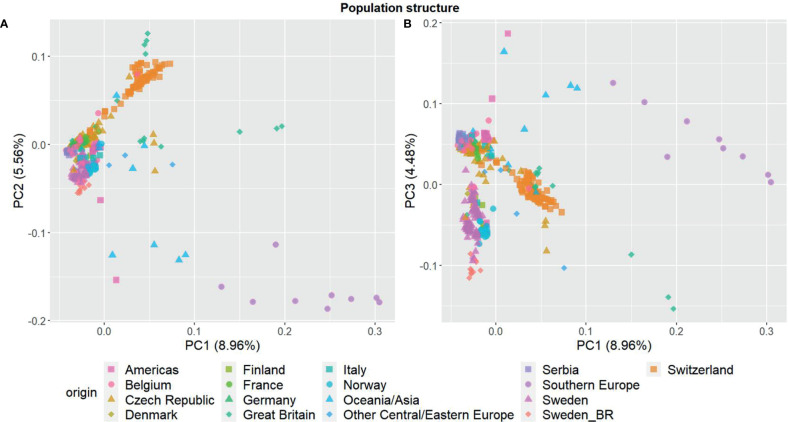
Principal component analysis showing the population structure among the 392 red clover (*Trifolium pratense*) accessions based on 20, 156 filtered and imputed SNPs. **(A)** first and second principal component; **(B)** first and third component. Accessions are grouped by country or region of origin. F2 breeding populations from Sweden (Sweden_BR) were kept as a separate group.

**Figure 3:**
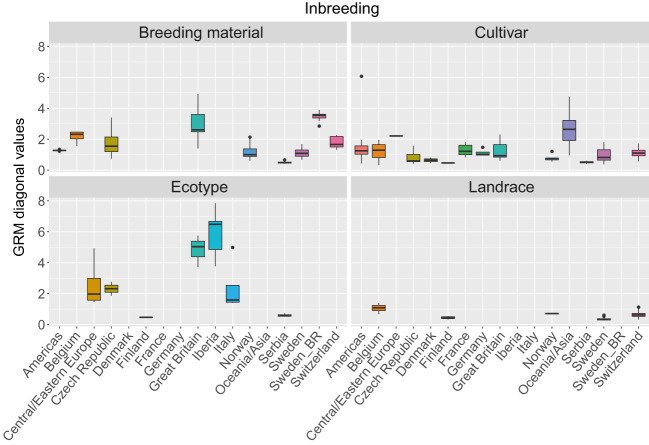
Variation in inbreeding expressed as the as values of the diagonal of the genomic relationship matrix (GRM) among 392 red clover accessions from different countries or regions. The GRM was calculated based on SNPs allele frequencies. Values above 1 indicate inbreeding, while values below 1 indicate outbreeding. F2 breeding populations from Sweden (Sweden_BR) were kept as a separate group. See **Table 1** for number of accessions belonging to each geographical group.

The three first principal components of the PCA (**Figure 2**) together explained 19% of the genetic variation and indicated that some of the genetic differentiation among the accessions in this study is associated with their origin. Accessions from Iberia were distinct from the other accessions but also diverse. Accessions from some other countries or regions were also relatively diverse (e.g. Great Britain and Czech Republic), while other groups were less diverse (e.g. Switzerland, Norway and Serbia). The genomic relationship values between populations ranged between -0.66 and 6.94 for SNPs and -0.66 and 7.38 for haplotypes, with median values close to zero (-0.03) for both (data not shown). The diagonal elements of the GRMs ranged between 0.25 and 7.84 for the SNPs and between 0.27 and 8.25 for the haplotypes, with median values of 0.92 and 0.97, respectively. The mean diagonal element varied between groups of accessions (**Figure 3**). Ecotypes from Iberia and Great Britain had the highest values (5-6.5), followed by cultivars from Oceania/Asia and breeding material from Great Britain and Sweden (2.5-3.5), reflecting inbreeding. Almost all landraces had GRM-values below 1, indicating outbreeding, while cultivars from most countries had mean values close to 1 or slightly less.

### 3.3 SNP- and haplotype-based GWAS

**Figure 4:**
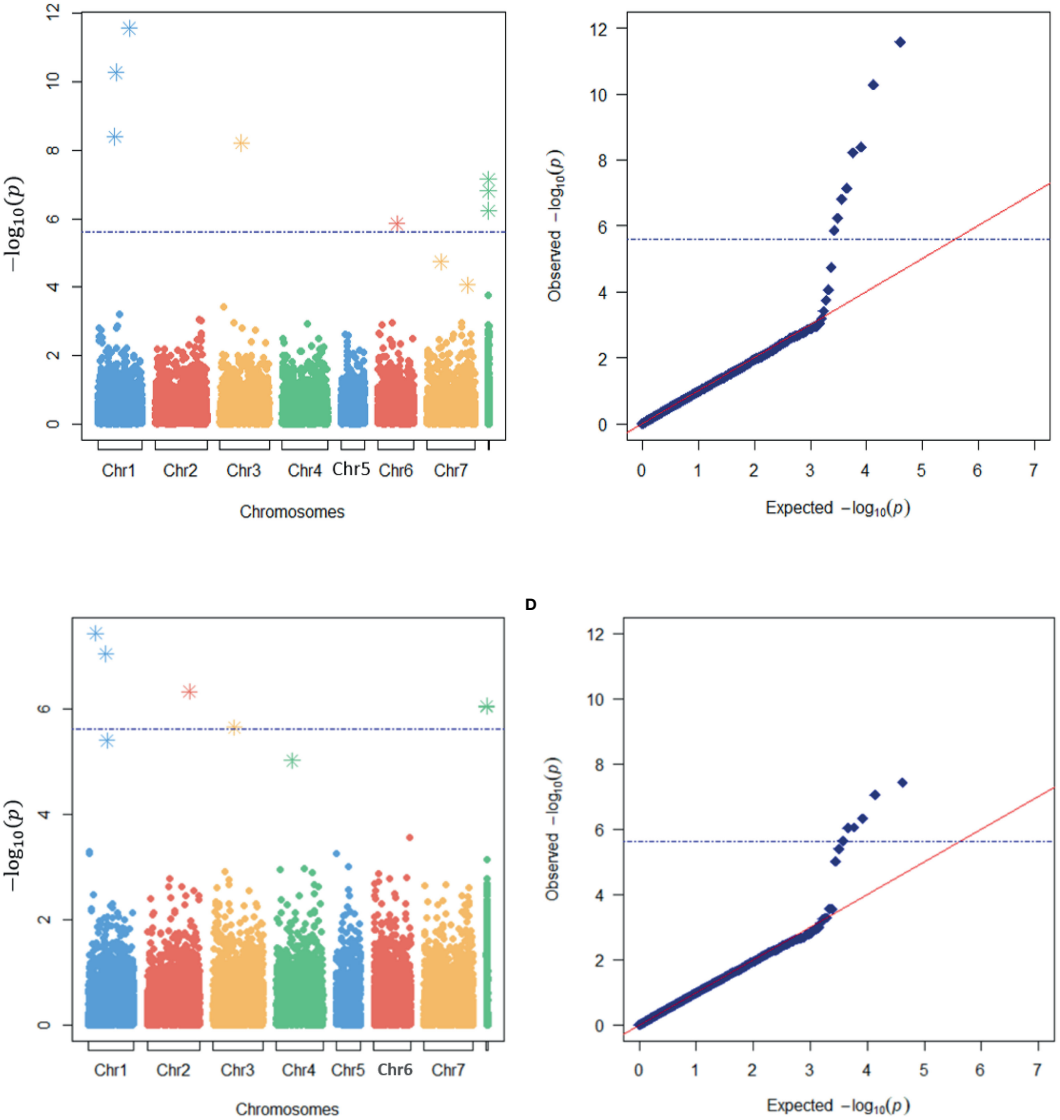
Identification of markers associated with freezing tolerance in a genome-wide association study (GWAS) of red clover. Manhattan **(A, C)** and quantile-quantile (QQ) **(B, D)** plots of the expected and observed -log10(p) –values of markers in the SNP-based **(A, B)** and haplotype-based **(C, D)** GWAS models with the lowest e-BIC values. The blue broken horizontal lines represent the Bonferroni threshold while asterisks represent the SNPs or haplotypes used as cofactors in the MLMM analyses.

A total of eight and six loci were detected as significantly associated with LT50 by the SNP- and haplotype-based GWAS, respectively (**Figure 4**). One significant SNP was located within one significant HTP, *i.e.*, a total of thirteen significant GBS loci were identified (**Table 3**). Eight were located on chromosomes while five were located on unanchored scaffolds. One more haplotype marker on LG1 was just below the Bonferroni threshold of significance, but clearly separated from the expected distribution of p-values in the quantile-quantile (QQ) plot (**Figure 4B**). This HTP also contained one of the significant SNPs.

**Table 3:** Phenotypic variance in LT50 explained (*R* ^2^) by significant markers identified in GWAS.

A			
Model	*R* ^2^ with kinship	*R* ^2^ without kinship	Number of loci
SNP-based	0.30	0.48	8
Haplotype-based	0.26	0.45	7^1^
B			
Marker	*R* ^2^ with kinship	*R* ^2^ without kinship	
SNP-based model			
LG1_10733810	0.05	0.09	
LG1_11608488	0.05	0.19	
scaf_836_20601	0.05	0.10	
scaf_1414_9867	0.04	0.01	
LG1_20019082	0.02	0.08	
scaf_153_142327	0.02	0.00	
LG6_12095124	0.01	0.00	
LG3_13453807	0.01	0.12	
Haplotype-based model			
LG1:11608425-11608558_3^1,2^	0.05	0.19	
LG1:10733751-10733981_31	0.05	0.09	
LG2:26124549-26124674_01	0.04	0.15	
LG1:4339801-4340029_24	0.04	0.03	
LG3:13143867-13144034_06	0.04	0.12	
scaf_670:26744-26998_19	0.03	0.08	
scaf_28637:389-568_03	0.02	0.03	

Regression analysis was performed, using allele frequencies of significant markers as predictors of LT50. The *R* ^2^ is given for both SNP- and haplotype-based models that either account for or do not account for kinship among accessions. A, *R* ^2^ for the regression models including all markers simultaneously; B, *R* ^2^ for the regression models including single markers. ^1^One haplotype had a p-value below the Bonferroni threshold of significance in the GWAS but it was included in further analyses because it was very close to being significant and included one significant SNP. ^2^Numbers at the end of each haplotype correspond to the variant number within HTP loci.

When kinship was accounted for, the phenotypic variance explained (R^2^) by the GWAS models with the lowest e-BIC values was 0.30 and 0.26 for the SNP- and haplotype-based analysis, respectively. When kinship was not accounted for, these values were 0.48 and 0.45 (**Table 3A**). The values for the individual significant SNPs, when kinship was accounted for ranged from 0.01 to 0.05, while those for the individual haplotypes ranged from 0.02 to 0.05 (**Table 3B**).

Some of the significant loci were characterized by a relatively strong effect of kinship (**Table 3B**; **Supplementary Figures 7–10**). Not surprisingly, these loci were also those whose distribution of allele frequencies was clearly associated with the origin of the plant material (**Supplementary Figure 7**).

### 3.4 Candidate gene search

For nine of the thirteen loci that were significantly associated with FT, the marker was located within a gene, for one locus the marker was located in close proximity (<0.5 kb) of a gene, while for the remaining three loci the marker was located on scaffolds that contained no genes. Functional annotations and the best BLAST hits to the *A. thaliana* or *M. truncatula* genome are reported in **Table 4**; **Supplementary Table 2**, respectively.

**Table 4:** Genes containing significant SNPs and/or haplotypes identified by GWAS of freezing tolerance (FT) in red clover.

SNP and/or haplotype	Annotation	Putative role in FT
LG1_10733810^1^ LG1:10733751-10733981	caffeoyl shikimate esterase	Lignin synthesis, secondary cell wall strengthening
LG1_11608488 LG1:11608425-11608558^2^	inositol transporter 1	Transport of compatible solutes, cellular signaling
scaf_836_20601	ATP/DNA binding protein	
LG1:4339801-4340029	cationic peroxidase 1	Oxidative stress response, lignin synthesis
LG3:13143867-13144034	transducin/WD40 repeat-like superfamily protein (NEDD1)	Microtubule organization, cellular signaling and trafficking
LG2:26124549-26124674	chloride channel protein CLC-c	
LG1_20019082	cationic amino acid transporter 1	Amino acid metabolism, accumulation and signaling
scaf_153_142327	NETWORKED 1A	Actin-binding, cellular signaling and trafficking
LG6_12095124	sucrose transport protein SUC8	Sucrose metabolism, accumulation and signaling
LG3_13453807	3-hydroxyisobutyryl-CoA hydrolase 1	Cold stress signaling, valine catabolism

Further details on BLASTn hits in the *A. thaliana* and *M. truncatula* genomes are given in **Supplementary Table 2**. Three of the scaffolds containing significant markers (not shown) were very short and contained no genes. ^1^ SNP located 80 bp outside the gene; ^2^haplotype marker had p-value slightly below the Bonferroni threshold of significance.

## 4 Discussion

### 4.1 Phenotypic and genotypic variation

There was considerable phenotypic variation in FT among the studied red clover accessions, with LT50 values ranging from –6.0 to –11.5°C. For comparison, 48 Nordic gene bank accessions and 6 Nordic cultivars tested using the same protocol ranged from -6.7 to -13.1°C (Zanotto et al., 2021), while a mixture of northern Fennoscandian breeding material given a much longer cold acclimation treatment had an LT50 of –15.1°C (Dalmannsdóttir et al., 2016). Bertrand et al. (2020) found LT50 values of –8 to -10°C in two Canadian cultivars acclimated under semi-natural conditions over the whole autumn and winter, and after recurrent selection for FT it increased to –15°C. The group consisting of nine accessions from the Iberian peninsula contained the most freezing sensitive accessions, but also some that were relatively freezing tolerant. According to the PCA this group was also both distinct from and more diverse than the other groups.

Red clover is known to be a genetically and phenotypically diverse crop (Nay et al., 2023). Ergon et al. (2022), studying GBS loci of a single cultivar, found an average of 3.6 haplotype variants per HTP, both when *Pst*I and *ApeK*I were used as restriction enzymes. In the present study, using similar calling and filtering criteria, we found only 2.8 haplotype variants per HTP on average. The true haplotype diversity is likely to be higher, but as the sequencing depth and consequently the SNP density was much lower in the present study (~20,000 vs. ~100,000 SNPs), and because a haplotype must be present in many accessions to reach a MAF > 0.05, a large number of rare polymorphisms are likely to be either not detected or filtered out. This implies that we here explore the genetic variation defined by the most common markers and are likely to miss variation that is due to less common markers. The extremely low LD that we observed is typical in outbreeding species with a high genetic diversity and in line with those found previously in studies considering wide and diversified plant material of red clover (Jones et al., 2020).

The expected diagonal values of the GRMs (relationship of each accession to itself) are 1.0 plus the inbreeding coefficient (Ashraf et al., 2016). The higher average values for the diagonal element of the GRMs that we found in some groups of accessions may be due to some level of inbreeding within those accessions. Some of the accessions used in this study are cultivars and breeding populations originating from a limited number of parents. Often these parents are chosen from already released cultivars within breeding programs which may share the same allele at several loci, and therefore have a reduced level of diversity within these loci. In this study, this was probably the case for breeding material from Sweden, Belgium and Great Britain and cultivars from Asia which all had mean GRM diagonal values exceeding 2. Another likely cause for the inbreeding that we observe in breeding material is the selection for specific traits, which also results in lower diversity and heterozygosity. Interestingly, high mean GRM diagonal values were also found among ecotypes from Iberia and Great Britain and to a lesser degree among those from Czech Republic, while ecotypes from Serbia and Finland had values well below 1, indicating outbreeding. Ecotypes that are located within restricted and isolated areas may have an increased level of inbreeding while other ecotypes may be part of large outbreeding populations.

### 4.2 GWAS and the proportion of phenotypic variation explained by significant loci

Population allele frequency data from pools have been successfully used in identifying loci associated with specific traits and for the development of genomic prediction models in perennial and Italian ryegrass (*L. multiflorum*) and red clover (Fè et al., 2015; Fè et al., 2016; Guo et al., 2018; Knorst et al., 2019; Ergon et al., 2019; Keep et al., 2020; Ergon et al., 2022; Frey et al., 2022), but this is to our knowledge the first study that uses GBS-generated allele frequency data from population pools to conduct haplotype-based GWAS in such outbred forage species. Our analyses confirmed that haplotype information can reveal associations that are not detectable by single SNPs and *vice versa*, as previously shown by Lorenz et al. (2010); Hamblin and Jannink (2011); Bekele et al. (2018) and Ergon et al. (2022).

With the strong LD decay observed, we would need at least a million polymorphic loci or so to be able to detect all loci with an effect on FT. Thus, with the ~20 000 SNPs in around 5000 HTP loci in this study, only a fraction of the loci can be expected to be found. It may therefore be surprising that as much as 30% and 26% of the phenotypic variation could be explained by the loci identified in the SNP- and haplotype-based models, respectively. A likely explanation for this is that variation in these loci correlates with variation in a lot of other loci controlling FT. Many other loci are likely to also have been under selection towards better FT in populations frequently exposed to freezing temperatures, and the loci we identified in the current study may therefore also explain some of the phenotypic variance caused by these. Since this study is performed at population level, it will only capture variation between, and not within, accessions.

For the markers LG3_13453807, LG1_11608488 and LG1:11608425-11608558, accessions from Iberia (characterized by low average FT) had a clearly different average allele frequency than those from the other countries, suggesting that the former material has a very low frequency for an allele which likely provides a better level of FT. Interestingly, both GBS loci which were shared across SNPs and haplotype-based GWAS (LG1_11608488 and LG1:11608425-11608558; LG1_10733810 and LG1:10733751-10733981) were among the loci with the highest *R* ^2^ from a regression model with LT50, which show that these associations are particularly strong. The same loci also had a large difference between from regressions that did and did not include kinship, being therefore strongly associated with the relatedness among accessions. However, even after accounting for the effect of kinship these loci could explain a significantly large proportion of the phenotypic variation.

### 4.3 Candidate genes

Because of the very rapid LD decay we considered only the closest gene to significant loci as candidate genes, since the likelihood of markers located further away being in LD with FT-associated alleles is low. Ten of the GBS loci significantly associated with variation of FT that mapped within gene sequences, and two more loci were found very close to a gene (< 0.5kb).

LG1_10733810, one of the three markers which could explain the largest phenotypic variance (5% after correcting for kinship), is located in a gene encoding caffeoyl shikimate esterase. This enzyme is essential for lignin synthesis and secondary cell wall formation in *M. truncatula* (Ha et al., 2016), which may play a role in cold acclimation and freezing tolerance through an effect of cell wall strengthening (Sasidharan et al., 2011). Additionally, some secondary metabolites synthesized through the shikimate pathway were reported to be involved in cold acclimation in *A. thaliana* and red clover (Schulz et al., 2016; Zanotto et al., 2023). Allele frequencies of caffeoyl shikimate esterase were moderately associated with kinship. The SNP allele associated with improved FT was mainly found among Nordic and Czech accessions while it was almost absent among Iberian accessions.

LG1_11608488 is located in a gene encoding an inositol transporter 1 (INT1) protein. INT1 transports *myo*-inositol from the vacuole into the cytoplasm in *A. thaliana* (Schneider et al., 2008; Strobl et al., 2018). Inositol transporters and *myo*-inositol have important roles in various signaling pathways in plants (Valluru and Van den Ende, 2011; Zhou et al., 2021). The methylated derivatives of myo-inositol (e.g. pinitol) accumulate in different plant species in response to abiotic stress, and is thought to act as an osmo-regulator under abiotic stresses (Sengupta et al., 2008; Sengupta et al., 2012). Pinitol concentration in red clover crowns increases during cold acclimation, and to higher levels in freezing tolerant material of different genetic backgrounds, supporting a role in freezing tolerance in red clover (Bertrand et al., 2020; Zanotto et al., 2023). It also increases in red clover in response to drought (Yates et al, 2014). Allele frequencies of this marker were strongly associated with kinship and markedly different in Iberian vs other groups of accessions. However, after correcting for kinship it still explained 5% of the phenotypic variation in FT in our study.

One candidate gene encodes a peroxidase, which may be involved in both lignin synthesis and/or oxidative stress response. Plants exposed to low temperatures combined with light are subjected to oxidative stress because of an energy imbalance in the photosystem (Huner et al., 1993). Several of the other identified candidate genes encode proteins that are involved in actin binding, microtubule organization, amino acid and carbohydrate metabolism.

### 4.4 Concluding remarks

Considering the current and future need for a more self-sufficient and sustainable production of plant-based protein in Europe, the availability of red clover cultivars with improved persistency is of primary importance to guarantee higher and more stable yields for this species. We have here described the variation in FT across a broad European collection of red clover accessions and identified loci and possible candidate genes associated with this trait. The genetic polymorphisms that were identified may, after validation in relevant germplasm, be transformed into molecular markers and used in selection for this trait. Furthermore, the integration of these markers with markers related to disease resistance and other traits of interest in a genomic selection model will contribute to a powerful tool to accelerate breeding of improved red clover.

## Data availability statement

The original contributions presented in the study are publicly available. This data can be found here: NMBU Open Research Data https://dataverse.no/dataset.xhtml?persistentId=doi:10.18710/WF1AGU. The GBS reads are available at NCBI under project number PRJNA842231.

## Author contributions

CG and RK were responsible for establishing a comprehensive red clover germplasm collection and for collecting and distributing red clover seeds. LS was responsible for DNA extractions and genotyping. LS and TR designed the genotyping strategy. TR performed SNP and haplotype calling. ÅE designed the phenotyping strategy and SZ performed the phenotyping. SZ performed all analyses of phenotypic and marker data with supervision from MP and ÅE. SZ wrote the paper with supervision from ÅE. All authors contributed to the article and approved the submitted version.

## Footnotes

1 www.eucleg.eu

2 www.lgcgroup.com

3 www.legumeinfo.org
