# Supplementary material for: A genome-wide association study of freezing tolerance in red clover (Trifolium pratense L.) germplasm of European origin
Source: Front Plant Sci. 2023 May 10;14:1189662. doi: 10.3389/fpls.2023.1189662 (PMC10208120; doi:10.3389/fpls.2023.1189662)
Supplement: Supplementary file 1 [file DataSheet_1.docx]

Supplementary Material

**A genome-wide association study of freezing tolerance in red clover (*Trifolium pratense* L.) germplasm of European origin**

**Stefano Zanotto^1^, Tom Ruttink^2^, Marie Pegard^3^, Leif Skøt^4^, Christoph Grieder^5^, Roland Kӧlliker^6^ and Åshild Ergon^1*^**

*** Correspondence:** Corresponding Author: ashild.ergon@nmbu.no

**Supplementary Material 1.** SNP calling procedure and allele frequency estimation

Reads were demultiplexed with cutadapt 3.3 (Martin, 2011), and 3’ restriction site remnants, common adapter sequences and 5’ restriction site remnants were removed using a custom python script, cutadapt 3.3 (Martin, 2011) and FASTX-Toolkit 0.0.14 (Gordon & Hannon, 2010). All reads were merged with a minimum overlap of 10 bp (PEAR, Zhang et al., 2014). Merged reads were quality filtered and reads shorter than 60 bp were discarded. Reads were aligned to the red clover reference genome sequence v2.1 (De Vega et al., 2015) with the BWA-mem algorithm implemented in BWA 0.7.17 (Li & Durbin, 2009) with default parameters. Alignments were sorted, indexed and filtered on mapping quality 20 (q20) with SAMtools 1.10 (Li et al., 2009).

Mpileup files (converted from BAM format with SAMtools) in which all genome positions with minimum read depth of 30 were concatenated, thus joining the neighboring GBS stacks and excluding the part of the genome without coverage, were used as input to calculate Watterson’s θ estimator with NPStat v0.99 (Ferretti et al., 2013). NPStat was run with the following settings: minor allele count equal to one read (MAC1), window-size equal to 10,000 bp, haploid sample size equal to 120 (the maximum number technically acceptable in NPStat), and maximum coverage equal to 500. Per population, a single genome-wide θ value was calculated as the mean across all windows (about 500 windows per sample). Loci with very high (>500) read depth may be derived from repetitive sequences that are mapped onto a single genomic locus and were thus excluded.

The NPStat-derived theta values per pool-GBS sample were used as diversity prior to the Bayesian SNP calling algorithm implemented in SNAPE-pooled (Raineri et al., 2012) to identify significant SNPs in each population. SNAPE-pooled was run with the following settings: - priortype = informative, -fold = folded, -nchr = 120 for consistency with NPStat.

The relative proportion of reads per SNP allele was used as an estimate of allele frequency. We used a custom python script to apply filters on the SNAPE-pooled reference allele frequency data. Filters were applied in the following order: (i) SNP positions were deleted if the reference allele was not A, C, G, or T, (ii) SNP frequencies were set to missing data when the two observed alleles were both different from the reference allele, or when the sum of the reference and the alternative allele read counts was lower than 27, (iii) using the Bayesian estimates of allele presence provided by SNAPE-pooled, we set the alternative allele frequency (and allele counts) to 0 if p(freq_alt_≠0) < 0.95 and the reference allele frequency (and allele counts) to 0 if p(freq_ref_≠0) < 0.95, (iv) after all the above filtering, we discarded SNP positions with more than two remaining alleles across the whole set of populations (thus removing potential residual low frequency sequencing errors).

**References**

De Vega JJ, Ayling S, Hegarty M, et al. (2015). Red clover (Trifolium pratense L.) draft genome provides a platform for trait improvement. Scientific Reports 5:17394. doi: 10.1038/srep17394

Ferretti L, Ramos-Onsins SE, PérezPerez-Enciso M. (2013). Population genomics from pool sequencing. Mol Ecol. 22(22):5561–5576. doi: 10.1111/mec.12522

Gordon A, Hannon G. (2010). Fastx-toolkit. FASTQ/A short-reads preprocessing tools (unpublished).

Li H, Durbin R. (2009). Fast and accurate short read alignment with Burrows–Wheeler transform. Bioinformatics. 25(14):1754–1760. doi 10.1093/bioinformatics/btp324

Li H, Handsaker B, Wysoker A, Fennell T, Ruan J, Homer N, et al. (2009). The sequence alignment/map format and SAMtools. Bioinformatics. 25(16):2078–2079. doi: 10.1093/bioinformatics/btp352

Martin M. (2011). Cutadapt removes adapter sequences from high-throughput sequencing reads. EMBnet journal. 17(1):10–12. doi: 10.14806/ej.17.1.200.

Raineri E, Ferretti L, Esteve-Codina A, Nevado B, Heath S, Pérez-Enciso M. (2012). SNP calling by sequencing pooled samples, BMC Bioinformatics,13:239, doi: 10.1186/1471-2105-13-239

Zhang J, Kobert K, Flouri T, & Stamatakis A. (2014). PEAR: A fast and accurate Illumina Paired End reAd mergeR. Bioinformatics, 30(5):614–620. doi: 10.1093/bioinformatics/btt593

**Supplementary Tables**

**Supplementary Table 1.** Information about the red clover accessions phenotyped for freezing tolerance. For each accession the ID from the EUCLEG project, the name, the providing institution, the origin and the type of material is indicated.

| **Accession ID** | **Accession Name** | **Institution** | **Country of origin** | **Type** |
| --- | --- | --- | --- | --- |
| EUC_TP_001 | Dimanche | INRA, FRA | FRA | Cultivar |
| EUC_TP_002 | Discovery | INRA, FRA | FRA | Cultivar |
| EUC_TP_003 | Formica | Agroscope, CHE | CHE | Cultivar |
| EUC_TP_004 | Milvus | Agroscope, CHE | CHE | Cultivar |
| EUC_TP_005 | Pavo | Agroscope, CHE | CHE | Cultivar |
| EUC_TP_006 | S586 AberClaret | IBERS, GBR | GBR | Cultivar |
| EUC_TP_007 | S592 AberChianti | IBERS, GBR | GBR | Cultivar |
| EUC_TP_008 | Gandalf | Graminor, NOR | NOR | Cultivar |
| EUC_TP_009 | Lea | Graminor, NOR | NOR | Cultivar |
| EUC_TP_010 | K 17 | IKBKS, SRB | SRB | Cultivar |
| EUC_TP_011 | K 38 | IKBKS, SRB | SRB | Cultivar |
| EUC_TP_012 | SW Ares | Lantmännen, SWE | SWE | Cultivar |
| EUC_TP_013 | Cyllene | DLF Seeds, CZE | CZE | Cultivar |
| EUC_TP_014 | Himalia | DLF Seeds, CZE | CZE | Cultivar |
| EUC_TP_015 | Metis | DLF Seeds, CZE | CZE | Cultivar |
| EUC_TP_016 | SANGRIA | RAGT2n, FRA | FRA | Cultivar |
| EUC_TP_017 | Saija | Boreal, FIN | FIN | Cultivar |
| EUC_TP_018 | Global | ILVO, BEL | BEL | Cultivar |
| EUC_TP_019 | Merviot | ILVO, BEL | BEL | Cultivar |
| EUC_TP_020 | Bonus | Agricultural Res. Ltd., CZE | CZE | Cultivar |
| EUC_TP_021 | NGB1132 | NordGen, SWE | FIN | Landrace |
| EUC_TP_022 | NGB1133 | NordGen, SWE | FIN | Landrace |
| EUC_TP_023 | NGB1142 | NordGen, SWE | FIN | Landrace |
| EUC_TP_024 | NGB14322 | NordGen, SWE | FIN | Ecotype |
| EUC_TP_025 | NGB1730 | NordGen, SWE | DNK | Cultivar |
| EUC_TP_026 | NGB2161 | NordGen, SWE | NOR | Landrace |
| EUC_TP_027 | NGB2391 | NordGen, SWE | SWE | Landrace |
| EUC_TP_028 | NGB2392 | NordGen, SWE | SWE | Landrace |
| EUC_TP_029 | NGB2458 | NordGen, SWE | SWE | Landrace |
| EUC_TP_030 | NGB2461 | NordGen, SWE | SWE | Landrace |
| EUC_TP_031 | NGB2487 | NordGen, SWE | SWE | Landrace |
| EUC_TP_032 | NGB2490 | NordGen, SWE | SWE | Landrace |
| EUC_TP_033 | NGB2492 | NordGen, SWE | SWE | Landrace |
| EUC_TP_034 | NGB4089 | NordGen, SWE | SWE | Landrace |
| EUC_TP_035 | Grasslands Colenso | PGG Wrightson, NZL | NZL | Cultivar |
| EUC_TP_036 | Sensation | PGG Wrightson, NZL | NZL | Cultivar |
| EUC_TP_037 | Affoltern i.E._328 | Agroscope, CHE | CHE | Landrace |
| EUC_TP_038 | Affoltern i.E._6 | Agroscope, CHE | CHE | Landrace |
| EUC_TP_039 | Belpberg_225 | Agroscope, CHE | CHE | Landrace |
| EUC_TP_040 | Belpberg_226 | Agroscope, CHE | CHE | Landrace |
| EUC_TP_041 | Belpberg_229 | Agroscope, CHE | CHE | Landrace |
| EUC_TP_042 | Bern_78 | Agroscope, CHE | CHE | Landrace |
| EUC_TP_043 | Bowil_119 | Agroscope, CHE | CHE | Landrace |
| EUC_TP_044 | Bruetten_3 | Agroscope, CHE | CHE | Landrace |
| EUC_TP_045 | Bubikon_8 | Agroscope, CHE | CHE | Landrace |
| EUC_TP_046 | Burgistein_300 | Agroscope, CHE | CHE | Landrace |
| EUC_TP_047 | Columba | Agroscope, CHE | CHE | Cultivar |
| EUC_TP_048 | Corvus | Agroscope, CHE | CHE | Cultivar |
| EUC_TP_049 | Dafila | Agroscope, CHE | CHE | Cultivar |
| EUC_TP_050 | Frauenkappelen_86 | Agroscope, CHE | CHE | Landrace |
| EUC_TP_051 | Goldbach i.E._167 | Agroscope, CHE | CHE | Landrace |
| EUC_TP_052 | Huttwil_50 | Agroscope, CHE | CHE | Landrace |
| EUC_TP_053 | Huttwil_60 | Agroscope, CHE | CHE | Landrace |
| EUC_TP_054 | Koeniz_231 | Agroscope, CHE | CHE | Landrace |
| EUC_TP_055 | Koeniz_279 | Agroscope, CHE | CHE | Landrace |
| EUC_TP_056 | Krauchthal_176 | Agroscope, CHE | CHE | Landrace |
| EUC_TP_057 | Lanzenhaeusern_291 | Agroscope, CHE | CHE | Landrace |
| EUC_TP_058 | Lestris | Agroscope, CHE | CHE | Cultivar |
| EUC_TP_059 | Merula | Agroscope, CHE | CHE | Cultivar |
| EUC_TP_060 | Milonia | Agroscope, CHE | CHE | Cultivar |
| EUC_TP_061 | Monaco | Agroscope, CHE | CHE | Cultivar |
| EUC_TP_062 | Niederwangen_262 | Agroscope, CHE | CHE | Landrace |
| EUC_TP_063 | Niederwangen_75 | Agroscope, CHE | CHE | Landrace |
| EUC_TP_064 | Oberthal_121 | Agroscope, CHE | CHE | Landrace |
| EUC_TP_065 | Oberuzwil_280 | Agroscope, CHE | CHE | Landrace |
| EUC_TP_066 | Pastor | Agroscope, CHE | CHE | Cultivar |
| EUC_TP_067 | Riedbach_88 | Agroscope, CHE | CHE | Landrace |
| EUC_TP_068 | Rueegsau_160 | Agroscope, CHE | CHE | Landrace |
| EUC_TP_069 | Rueti_314 | Agroscope, CHE | CHE | Landrace |
| EUC_TP_070 | Schmidigen_336 | Agroscope, CHE | CHE | Landrace |
| EUC_TP_071 | Semperina | Agroscope, CHE | CHE | Cultivar |
| EUC_TP_072 | Signau_140 | Agroscope, CHE | CHE | Landrace |
| EUC_TP_073 | Sumiswald_189 | Agroscope, CHE | CHE | Landrace |
| EUC_TP_074 | Ueberstorf_294 | Agroscope, CHE | CHE | Landrace |
| EUC_TP_075 | Ueberstorf_346 | Agroscope, CHE | CHE | Landrace |
| EUC_TP_076 | Ufhusen_52 | Agroscope, CHE | CHE | Landrace |
| EUC_TP_077 | Uttigen_2 | Agroscope, CHE | CHE | Landrace |
| EUC_TP_078 | Wynigen_335 | Agroscope, CHE | CHE | Landrace |
| EUC_TP_079 | Zaeziwil_125 | Agroscope, CHE | CHE | Landrace |
| EUC_TP_080 | Zaeziwil_127 | Agroscope, CHE | CHE | Landrace |
| EUC_TP_081 | Aa 3100 | IBERS, GBR | GBR | Old cultivar |
| EUC_TP_082 | Aa 3148 | IBERS, GBR | GBR | Old cultivar |
| EUC_TP_083 | Aa 3149 | IBERS, GBR | GBR | Old cultivar |
| EUC_TP_084 | AA 32 | IBERS, GBR | GBR | Breeder's Line |
| EUC_TP_085 | Aa 3459 | IBERS, GBR | GBR | Old cultivar |
| EUC_TP_086 | Aa 4190 | IBERS, GBR | POL | Ecotype |
| EUC_TP_087 | Aa 4292 | IBERS, GBR | CZE | Ecotype |
| EUC_TP_088 | Aa 4298 | IBERS, GBR | SVK | Ecotype |
| EUC_TP_089 | Aa 4351 | IBERS, GBR | BGR | Ecotype |
| EUC_TP_090 | Aa 4379 Britta | IBERS, GBR | GBR | Cultivar |
| EUC_TP_091 | Aa 4390 | IBERS, GBR | PRT | Ecotype |
| EUC_TP_092 | Aa 4400 | IBERS, GBR | GBR | Ecotype |
| EUC_TP_093 | Aa 4444 | IBERS, GBR | ITA | Ecotype |
| EUC_TP_094 | Aa 4516 | IBERS, GBR | ESP | Ecotype |
| EUC_TP_095 | Aa 4519 | IBERS, GBR | ESP | Ecotype |
| EUC_TP_097 | Aa 4528 | IBERS, GBR | ESP | Ecotype |
| EUC_TP_098 | Aa 4939 | IBERS, GBR | NZL | Cultivar |
| EUC_TP_099 | Aa 5674 | IBERS, GBR | ARG | Cultivar |
| EUC_TP_100 | Aa 5675 | IBERS, GBR | ARG | Cultivar |
| EUC_TP_101 | Aa 5676 | IBERS, GBR | ARG | Cultivar |
| EUC_TP_102 | Aa 5677 | IBERS, GBR | ARG | Cultivar |
| EUC_TP_103 | Aa 5678 | IBERS, GBR | ARG | Cultivar |
| EUC_TP_104 | Aa 5746 Harmonie | IBERS, GBR | GBR | Cultivar |
| EUC_TP_105 | Aa4380 Altaswede | IBERS, GBR | CAN | Cultivar |
| EUC_TP_106 | S543 AberRuby | IBERS, GBR | GBR | Cultivar |
| EUC_TP_107 | Aa 4940 Broadway | IBERS, GBR | NZL | Cultivar |
| EUC_TP_108 | TP9525 | Agroscope, CHE | CHE | Breeding material |
| EUC_TP_109 | TP9645 | Agroscope, CHE | CHE | Breeding material |
| EUC_TP_110 | TP9445 | Agroscope, CHE | CHE | Breeding material |
| EUC_TP_111 | TP9735 | Agroscope, CHE | CHE | Breeding material |
| EUC_TP_112 | TP9315 | Agroscope, CHE | CHE | Breeding material |
| EUC_TP_113 | Gumpensteiner Rotklee | HBLFA, AUT | AUT | Old cultivar |
| EUC_TP_114 | Cinnamon Plus | USDA, USA | USA | Cultivar |
| EUC_TP_115 | DFRC11 | USDA, USA | USA | Breeding material |
| EUC_TP_116 | DFRC12 | USDA, USA | USA | Breeding material |
| EUC_TP_117 | DFRC13 | USDA, USA | USA | Breeding material |
| EUC_TP_118 | DFRC14 | USDA, USA | USA | Breeding material |
| EUC_TP_119 | DFRC15 | USDA, USA | USA | Breeding material |
| EUC_TP_120 | FF 9615 | USDA, USA | USA | Cultivar |
| EUC_TP_121 | Marathon | USDA, USA | USA | Cultivar |
| EUC_TP_122 | Starfire I | USDA, USA | USA | Cultivar |
| EUC_TP_123 | Starfire II | USDA, USA | USA | Cultivar |
| EUC_TP_124 | GnRk0729 | Graminor, NOR | NOR | Breeding material |
| EUC_TP_125 | GnRk0747 | Graminor, NOR | NOR | Breeding material |
| EUC_TP_126 | KvRk0201 | Graminor, NOR | NOR | Breeding material |
| EUC_TP_127 | LGRk8801 | Graminor, NOR | NOR | Breeding material |
| EUC_TP_128 | LGRk9415 | Graminor, NOR | NOR | Breeding material |
| EUC_TP_129 | Linus | Graminor, NOR | NOR | Cultivar |
| EUC_TP_130 | LøRk0286 | Graminor, NOR | NOR | Breeding material |
| EUC_TP_131 | LøRk0287 | Graminor, NOR | NOR | Breeding material |
| EUC_TP_132 | Linn | Graminor, NOR | NOR | Cultivar |
| EUC_TP_134 | LøRk9207 | Graminor, NOR | NOR | Cultivar |
| EUC_TP_135 | LøRk9625 | Graminor, NOR | NOR | Breeding material |
| EUC_TP_136 | LøRk9627 | Graminor, NOR | NOR | Breeding material |
| EUC_TP_137 | LøRk9628 | Graminor, NOR | NOR | Breeding material |
| EUC_TP_138 | LøRk9753 | Graminor, NOR | NOR | Breeding material |
| EUC_TP_139 | VåRk0401 | Graminor, NOR | NOR | Breeding material |
| EUC_TP_140 | VåRk0510 | Graminor, NOR | NOR | Breeding material |
| EUC_TP_141 | VåRk0512 | Graminor, NOR | NOR | Breeding material |
| EUC_TP_142 | VåRk0513 | Graminor, NOR | NOR | Breeding material |
| EUC_TP_143 | VåRk0624 | Graminor, NOR | NOR | Breeding material |
| EUC_TP_144 | VåRk0625 | Graminor, NOR | NOR | Breeding material |
| EUC_TP_145 | K 39 | IKBKS, SRB | SRB | Cultivar |
| EUC_TP_146 | Diplomat | DSV, DEU | DEU | Cultivar |
| EUC_TP_147 | SW 1479004 | Lantmännen, SWE | SWE | Breeding material |
| EUC_TP_148 | SW 1578301 | Lantmännen, SWE | SWE | Breeding material |
| EUC_TP_149 | SW 1678001 | Lantmännen, SWE | SWE | Breeding material |
| EUC_TP_150 | SW RK1092 | Lantmännen, SWE | SWE | Breeding material |
| EUC_TP_151 | SW RK1117 | Lantmännen, SWE | SWE | Breeding material |
| EUC_TP_152 | SW RK1118 | Lantmännen, SWE | SWE | Breeding material |
| EUC_TP_153 | SW RK1119 | Lantmännen, SWE | SWE | Breeding material |
| EUC_TP_154 | SW RK1120 | Lantmännen, SWE | SWE | Breeding material |
| EUC_TP_155 | SW RK1121 | Lantmännen, SWE | SWE | Breeding material |
| EUC_TP_156 | SW RK1122 | Lantmännen, SWE | SWE | Breeding material |
| EUC_TP_157 | SW RK1123 | Lantmännen, SWE | SWE | Breeding material |
| EUC_TP_158 | SW RK1124 | Lantmännen, SWE | SWE | Breeding material |
| EUC_TP_159 | SW RK1125 | Lantmännen, SWE | SWE | Breeding material |
| EUC_TP_160 | SW RK1131 | Lantmännen, SWE | SWE | Breeding material |
| EUC_TP_161 | SW RK1132 | Lantmännen, SWE | SWE | Breeding material |
| EUC_TP_162 | SW RK1133 | Lantmännen, SWE | SWE | Breeding material |
| EUC_TP_163 | SW RK1134 | Lantmännen, SWE | SWE | Breeding material |
| EUC_TP_164 | SW Yngve | Lantmännen, SWE | SWE | Cultivar |
| EUC_TP_165 | SWÅ RK09093 | Lantmännen, SWE | SWE | Breeding material |
| EUC_TP_166 | 0780MP2 | DLF Seeds, CZE | CZE | Breeding material |
| EUC_TP_167 | 08102MP2 | DLF Seeds, CZE | CZE | Breeding material |
| EUC_TP_168 | 08102MP4 | DLF Seeds, CZE | CZE | Breeding material |
| EUC_TP_169 | Callisto | DLF Seeds, CZE | CZE | Cultivar |
| EUC_TP_170 | Elara | DLF Seeds, CZE | CZE | Cultivar |
| EUC_TP_171 | Ganymed | DLF Seeds, CZE | CZE | Cultivar |
| EUC_TP_172 | Hegemon | DLF Seeds, CZE | CZE | Cultivar |
| EUC_TP_173 | Helike | DLF Seeds, CZE | CZE | Cultivar |
| EUC_TP_174 | HŽ 2004 80 – 01 | DLF Seeds, CZE | CZE | Breeding material |
| EUC_TP_175 | JL 2n 07 80 MP 1 | DLF Seeds, CZE | CZE | Breeding material |
| EUC_TP_176 | Kalyke | DLF Seeds, CZE | CZE | Cultivar |
| EUC_TP_177 | TPD-05-11-18002 | DLF Seeds, CZE | CZE | Breeding material |
| EUC_TP_178 | TPD-05-11-3087 | DLF Seeds, CZE | CZE | Breeding material |
| EUC_TP_179 | TPD-05-11-3088 | DLF Seeds, CZE | CZE | Breeding material |
| EUC_TP_180 | TPD-05-13-3080 | DLF Seeds, CZE | CZE | Breeding material |
| EUC_TP_181 | TPD-05-13-3085 | DLF Seeds, CZE | CZE | Breeding material |
| EUC_TP_182 | TPD-05-13-3091 | DLF Seeds, CZE | CZE | Breeding material |
| EUC_TP_183 | TPD-05-14-1011 | DLF Seeds, CZE | CZE | Breeding material |
| EUC_TP_184 | TPD-05-15-3127 | DLF Seeds, CZE | CZE | Breeding material |
| EUC_TP_185 | TPD-05-15-3128 | DLF Seeds, CZE | CZE | Breeding material |
| EUC_TP_186 | TPD-05-15-3129 | DLF Seeds, CZE | CZE | Breeding material |
| EUC_TP_187 | TPD-05-16-3076 | DLF Seeds, CZE | CZE | Breeding material |
| EUC_TP_188 | TPD-05-16-3146 | DLF Seeds, CZE | CZE | Breeding material |
| EUC_TP_189 | TPD-05-16-3177 | DLF Seeds, CZE | CZE | Breeding material |
| EUC_TP_190 | KARIM | RAGT2n, FRA | FRA | Cultivar |
| EUC_TP_191 | MISTRAL | RAGT2n, FRA | FRA | Cultivar |
| EUC_TP_192 | RAVVI | RAGT2n, FRA | FRA | Cultivar |
| EUC_TP_193 | TREVVIO | RAGT2n, FRA | FRA | Cultivar |
| EUC_TP_194 | Grasslands Hamua | AgResearch, NZ | NZL | Cultivar |
| EUC_TP_195 | Grasslands Turoa | AgResearch, NZ | NZL | Cultivar |
| EUC_TP_196 | Relish | AgResearch, NZ | NZL | Cultivar |
| EUC_TP_197 | Ruby/Enterprise | AgResearch, NZ | NZL | Cultivar |
| EUC_TP_198 | Natsuyu | Hokkaido Ag. Res., JPN | JPN | Cultivar |
| EUC_TP_199 | Ryokuyu | Hokkaido Ag. Res., JPN | JPN | Cultivar |
| EUC_TP_200 | NS-Mlava | IFVCNS, SRB | SRB | Cultivar |
| EUC_TP_201 | NS-Petnica | IFVCNS, SRB | SRB | Cultivar |
| EUC_TP_202 | NS-Sana | IFVCNS, SRB | SRB | Cultivar |
| EUC_TP_203 | Una(NS) | IFVCNS, SRB | SRB | Cultivar |
| EUC_TP_204 | Zoja (NS) | IFVCNS, SRB | SRB | Cultivar |
| EUC_TP_205 | Avisto | ILVO, BEL | BEL | Cultivar |
| EUC_TP_206 | Crossway | PGG Wrightson, NZL | NZL | Cultivar |
| EUC_TP_207 | Lemmon | ILVO, BE | BEL | Cultivar |
| EUC_TP_208 | Merkemse | ILVO, BE | BEL | Landrace |
| EUC_TP_209 | Tp.12.12 | ILVO, BE | BEL | Breeding material |
| EUC_TP_210 | Tp.14.7 | ILVO, BE | BEL | Breeding material |
| EUC_TP_211 | Tandy | ILVO, BE | BEL | Cultivar |
| EUC_TP_212 | Agil | Agricultural Res. Ltd., CZE | CZE | Cultivar |
| EUC_TP_214 | Brisk | Agricultural Res. Ltd., CZE | CZE | Cultivar |
| EUC_TP_215 | Chlumecký | Agricultural Res. Ltd., CZE | CZE | Cultivar |
| EUC_TP_218 | Feng | Agricultural Res. Ltd., CZE | CZE | Cultivar |
| EUC_TP_219 | Garant | Agricultural Res. Ltd., CZE | CZE | Cultivar |
| EUC_TP_223 | Respect | Agricultural Res. Ltd., CZE | CZE | Cultivar |
| EUC_TP_224 | Slavín | Agricultural Res. Ltd., CZE | CZE | Cultivar |
| EUC_TP_225 | Slavoj | Agricultural Res. Ltd., CZE | CZE | Cultivar |
| EUC_TP_227 | Spurt | Agricultural Res. Ltd., CZE | CZE | Cultivar |
| EUC_TP_228 | Start | Agricultural Res. Ltd., CZE | CZE | Cultivar |
| EUC_TP_229 | Suez | Agricultural Res. Ltd., CZE | CZE | Cultivar |
| EUC_TP_231 | Trubadur | Agricultural Res. Ltd., CZE | CZE | Cultivar |
| EUC_TP_232 | Van | Agricultural Res. Ltd., CZE | CZE | Cultivar |
| EUC_TP_233 | Vendelín | Agricultural Res. Ltd., CZE | CZE | Cultivar |
| EUC_TP_234 | Vltavín | Agricultural Res. Ltd., CZE | CZE | Cultivar |
| EUC_TP_236 | Zefyr | Agricultural Res. Ltd., CZE | CZE | Cultivar |
| EUC_TP_237 | Harmonie | NPZ, DEU | DEU | Cultivar |
| EUC_TP_238 | Regent | NPZ, DEU | DEU | Cultivar |
| EUC_TP_239 | NGB1736 | NordGen, SWE | DNK | Cultivar |
| EUC_TP_240 | NGB2347 | NordGen, SWE | SWE | Cultivar |
| EUC_TP_241 | NGB2349 | NordGen, SWE | SWE | Cultivar |
| EUC_TP_242 | NGB2395 | NordGen, SWE | SWE | Landrace |
| EUC_TP_243 | NGB2452 | NordGen, SWE | SWE | Landrace |
| EUC_TP_244 | NGB2453 | NordGen, SWE | SWE | Landrace |
| EUC_TP_245 | NGB2464 | NordGen, SWE | SWE | Landrace |
| EUC_TP_246 | NGB2465 | NordGen, SWE | SWE | Landrace |
| EUC_TP_247 | NGB2466 | NordGen, SWE | SWE | Landrace |
| EUC_TP_248 | NGB2468 | NordGen, SWE | SWE | Landrace |
| EUC_TP_249 | NGB2469 | NordGen, SWE | SWE | Landrace |
| EUC_TP_250 | NGB2471 | NordGen, SWE | SWE | Landrace |
| EUC_TP_251 | NGB2472 | NordGen, SWE | SWE | Landrace |
| EUC_TP_252 | NGB2473 | NordGen, SWE | SWE | Landrace |
| EUC_TP_253 | NGB2474 | NordGen, SWE | SWE | Landrace |
| EUC_TP_254 | NGB2475 | NordGen, SWE | SWE | Landrace |
| EUC_TP_255 | NGB2476 | NordGen, SWE | SWE | Landrace |
| EUC_TP_256 | NGB2477 | NordGen, SWE | SWE | Landrace |
| EUC_TP_257 | NGB2481 | NordGen, SWE | SWE | Landrace |
| EUC_TP_258 | NGB2482 | NordGen, SWE | SWE | Landrace |
| EUC_TP_259 | NGB2494 | NordGen, SWE | SWE | Landrace |
| EUC_TP_260 | NGB2495 | NordGen, SWE | SWE | Landrace |
| EUC_TP_261 | NGB2569 | NordGen, SWE | SWE | Landrace |
| EUC_TP_262 | NGB2598 | NordGen, SWE | SWE | Landrace |
| EUC_TP_263 | NGB2599 | NordGen, SWE | SWE | Landrace |
| EUC_TP_264 | NGB2600 | NordGen, SWE | SWE | Landrace |
| EUC_TP_265 | NGB2739 | NordGen, SWE | SWE | Cultivar |
| EUC_TP_266 | NGB2740 | NordGen, SWE | SWE | Cultivar |
| EUC_TP_267 | NGB2742 | NordGen, SWE | SWE | Cultivar |
| EUC_TP_268 | NGB2745 | NordGen, SWE | SWE | Cultivar |
| EUC_TP_269 | NGB2746 | NordGen, SWE | SWE | Cultivar |
| EUC_TP_270 | NGB2747 | NordGen, SWE | SWE | Cultivar |
| EUC_TP_271 | NGB2748 | NordGen, SWE | SWE | Cultivar |
| EUC_TP_272 | NGB2749 | NordGen, SWE | SWE | Cultivar |
| EUC_TP_273 | NGB2750 | NordGen, SWE | SWE | Cultivar |
| EUC_TP_274 | NGB2751 | NordGen, SWE | SWE | Cultivar |
| EUC_TP_275 | NGB4126 | NordGen, SWE | DNK | Cultivar |
| EUC_TP_277 | NGB7510 | NordGen, SWE | SWE | Cultivar |
| EUC_TP_278 | NGB9966 | NordGen, SWE | SWE | Landrace |
| EUC_TP_279 | Diadem | INRA, FRA | FRA | Cultivar |
| EUC_TP_280 | Diper | INRA, FRA | FRA | Old cultivar |
| EUC_TP_281 | Diplo | INRA, FRA | FRA | Cultivar |
| EUC_TP_282 | Kindia | INRA, FRA | FRA | Cultivar |
| EUC_TP_283 | Affoltern i.E._325 | Agroscope, CHE | CHE | Landrace |
| EUC_TP_284 | Bern_76 | Agroscope, CHE | CHE | Landrace |
| EUC_TP_285 | Bigenthal_163 | Agroscope, CHE | CHE | Landrace |
| EUC_TP_286 | Biglen_352 | Agroscope, CHE | CHE | Landrace |
| EUC_TP_287 | Englisberg_249 | Agroscope, CHE | CHE | Landrace |
| EUC_TP_288 | Grossdietwil_21 | Agroscope, CHE | CHE | Landrace |
| EUC_TP_289 | Haeusernmoos_333 | Agroscope, CHE | CHE | Landrace |
| EUC_TP_290 | Koeniz_239 | Agroscope, CHE | CHE | Landrace |
| EUC_TP_291 | Koeniz_247 | Agroscope, CHE | CHE | Landrace |
| EUC_TP_292 | Lauperswil_138 | Agroscope, CHE | CHE | Landrace |
| EUC_TP_293 | MontCalme | Agroscope, CHE | CHE | Old cultivar |
| EUC_TP_294 | Neuenegg_340 | Agroscope, CHE | CHE | Landrace |
| EUC_TP_295 | Niederscherli_273 | Agroscope, CHE | CHE | Landrace |
| EUC_TP_296 | Oberbottigen_7 | Agroscope, CHE | CHE | Landrace |
| EUC_TP_297 | Oberoenz_321 | Agroscope, CHE | CHE | Landrace |
| EUC_TP_298 | Oeschenbach_330 | Agroscope, CHE | CHE | Landrace |
| EUC_TP_299 | Renova | Agroscope, CHE | CHE | Old cultivar |
| EUC_TP_300 | Riggisberg_318 | Agroscope, CHE | CHE | Landrace |
| EUC_TP_301 | Rueedisbach_332 | Agroscope, CHE | CHE | Landrace |
| EUC_TP_302 | Rüttinova | Agroscope, CHE | CHE | Old cultivar |
| EUC_TP_303 | Schmitten_5 | Agroscope, CHE | CHE | Landrace |
| EUC_TP_304 | Signau_154 | Agroscope, CHE | CHE | Landrace |
| EUC_TP_305 | Wasen i.E._199 | Agroscope, CHE | CHE | Landrace |
| EUC_TP_306 | Weier i.E._327 | Agroscope, CHE | CHE | Landrace |
| EUC_TP_307 | Aa 3090 | IBERS, GBR | GBR | Old cultivar |
| EUC_TP_308 | Aa 3108 | IBERS, GBR | GBR | Old cultivar |
| EUC_TP_310 | Aa 4189 | IBERS, GBR | POL | Ecotype |
| EUC_TP_311 | Aa 4297 | IBERS, GBR | CZE | Ecotype |
| EUC_TP_312 | Aa 4398 | IBERS, GBR | GBR | Ecotype |
| EUC_TP_313 | Aa 4403 | IBERS, GBR | GBR | Ecotype |
| EUC_TP_315 | Aa 4445 | IBERS, GBR | ITA | Ecotype |
| EUC_TP_316 | Aa 4448 | IBERS, GBR | ITA | Ecotype |
| EUC_TP_317 | Aa 4456 | IBERS, GBR | ITA | Ecotype |
| EUC_TP_318 | Aa 4515 | IBERS, GBR | ESP | Ecotype |
| EUC_TP_319 | Aa 4520 | IBERS, GBR | ESP | Ecotype |
| EUC_TP_320 | Aa 4525 | IBERS, GBR | ESP | Ecotype |
| EUC_TP_321 | Aa 4527 | IBERS, GBR | ESP | Ecotype |
| EUC_TP_322 | Aa 4529 | IBERS, GBR | ESP | Ecotype |
| EUC_TP_324 | Aa 4593 | IBERS, GBR | GBR | Cultivar (Landrace) |
| EUC_TP_325 | Aa 4934 | IBERS, GBR | GBR | Breeder's Line |
| EUC_TP_326 | Aa 4936 | IBERS, GBR | GBR | Breeder's Line |
| EUC_TP_327 | Aa 4937 | IBERS, GBR | GBR | Breeder's Line |
| EUC_TP_328 | Aa 5417 | IBERS, GBR | GBR | Breeder's Line |
| EUC_TP_329 | SW 1479001 | Lantmännen, SWE | SWE | Breeding material |
| EUC_TP_330 | SW 1479002 | Lantmännen, SWE | SWE | Breeding material |
| EUC_TP_331 | SW 1479003 | Lantmännen, SWE | SWE | Breeding material |
| EUC_TP_332 | SW 1678002 | Lantmännen, SWE | SWE | Breeding material |
| EUC_TP_333 | SW 1678003 | Lantmännen, SWE | SWE | Breeding material |
| EUC_TP_334 | SW 1678004 | Lantmännen, SWE | SWE | Breeding material |
| EUC_TP_335 | SW 1678401 | Lantmännen, SWE | SWE | Breeding material |
| EUC_TP_336 | SW 1678402 | Lantmännen, SWE | SWE | Breeding material |
| EUC_TP_337 | SW 1678403 | Lantmännen, SWE | SWE | Breeding material |
| EUC_TP_338 | SW RK1095 | Lantmännen, SWE | SWE | Breeding material |
| EUC_TP_339 | SW RK1096 | Lantmännen, SWE | SWE | Breeding material |
| EUC_TP_340 | SW RK1097 | Lantmännen, SWE | SWE | Breeding material |
| EUC_TP_341 | SW RK1102 | Lantmännen, SWE | SWE | Breeding material |
| EUC_TP_342 | SW RK1160 | Lantmännen, SWE | SWE | Breeding material |
| EUC_TP_343 | SW RK1161 | Lantmännen, SWE | SWE | Breeding material |
| EUC_TP_344 | SW RK1162 | Lantmännen, SWE | SWE | Breeding material |
| EUC_TP_345 | SW RK1164 | Lantmännen, SWE | SWE | Breeding material |
| EUC_TP_346 | SWA 1376104 | Lantmännen, SWE | SWE | Breeding material |
| EUC_TP_347 | SWA 1376105 | Lantmännen, SWE | SWE | Breeding material |
| EUC_TP_348 | SWA 1476014 | Lantmännen, SWE | SWE | Breeding material |
| EUC_TP_349 | SWA 1476016 | Lantmännen, SWE | SWE | Breeding material |
| EUC_TP_350 | SWA 1476017 | Lantmännen, SWE | SWE | Breeding material |
| EUC_TP_351 | SWA 1476019 | Lantmännen, SWE | SWE | Breeding material |
| EUC_TP_352 | SWA 1575301 | Lantmännen, SWE | SWE | Breeding material |
| EUC_TP_353 | SWA 1575302 | Lantmännen, SWE | SWE | Breeding material |
| EUC_TP_354 | SWA 1575304 | Lantmännen, SWE | SWE | Breeding material |
| EUC_TP_355 | SWA 1575305 | Lantmännen, SWE | SWE | Breeding material |
| EUC_TP_356 | SWA 1575306 | Lantmännen, SWE | SWE | Breeding material |
| EUC_TP_357 | SWA 1575307 | Lantmännen, SWE | SWE | Breeding material |
| EUC_TP_358 | SWA 1575308 | Lantmännen, SWE | SWE | Breeding material |
| EUC_TP_359 | SWA 1575309 | Lantmännen, SWE | SWE | Breeding material |
| EUC_TP_360 | SWA 1575312 | Lantmännen, SWE | SWE | Breeding material |
| EUC_TP_361 | SWA 1576005 | Lantmännen, SWE | SWE | Breeding material |
| EUC_TP_362 | SWA 1675205 | Lantmännen, SWE | SWE | Breeding material |
| EUC_TP_363 | SWA 1675206 | Lantmännen, SWE | SWE | Breeding material |
| EUC_TP_364 | SWA 1675207 | Lantmännen, SWE | SWE | Breeding material |
| EUC_TP_365 | SWA 1675208 | Lantmännen, SWE | SWE | Breeding material |
| EUC_TP_366 | SWA 1675212 | Lantmännen, SWE | SWE | Breeding material |
| EUC_TP_367 | TPD-05-04-3000 | DLF Seeds, CZE | CZE | Breeding material |
| EUC_TP_368 | TPD-05-11-3007 | DLF Seeds, CZE | CZE | Breeding material |
| EUC_TP_369 | TPD-05-12-3018 | DLF Seeds, CZE | CZE | Breeding material |
| EUC_TP_370 | Avala (NS) | IFVCNS, SRB | SRB | Cultivar |
| EUC_TP_371 | BL-1-Banja Luka | IFVCNS, SRB | SRB | Breeding material |
| EUC_TP_372 | BL-3-Banja Luka | IFVCNS, SRB | SRB | Breeding material |
| EUC_TP_373 | BL-4-Banja Luka | IFVCNS, SRB | SRB | Breeding material |
| EUC_TP_374 | BL-5-Banja Luka | IFVCNS, SRB | SRB | Breeding material |
| EUC_TP_375 | D-1 | IFVCNS, SRB | SRB | Breeding material |
| EUC_TP_376 | D-10 | IFVCNS, SRB | SRB | Breeding material |
| EUC_TP_377 | D-2 | IFVCNS, SRB | SRB | Breeding material |
| EUC_TP_378 | D-3 | IFVCNS, SRB | SRB | Breeding material |
| EUC_TP_379 | D-4 | IFVCNS, SRB | SRB | Breeding material |
| EUC_TP_380 | D-5 | IFVCNS, SRB | SRB | Breeding material |
| EUC_TP_381 | D-6 | IFVCNS, SRB | SRB | Breeding material |
| EUC_TP_382 | D-7 | IFVCNS, SRB | SRB | Breeding material |
| EUC_TP_383 | D-8 | IFVCNS, SRB | SRB | Breeding material |
| EUC_TP_384 | D-9 | IFVCNS, SRB | SRB | Breeding material |
| EUC_TP_385 | M10-Kopaonik | IFVCNS, SRB | SRB | Ecotype |
| EUC_TP_386 | M11-Kopaonik | IFVCNS, SRB | SRB | Ecotype |
| EUC_TP_387 | M12-Kopaonik | IFVCNS, SRB | SRB | Ecotype |
| EUC_TP_388 | M13-Kopaonik | IFVCNS, SRB | SRB | Ecotype |
| EUC_TP_389 | M14-Kopaonik | IFVCNS, SRB | SRB | Ecotype |
| EUC_TP_390 | NS-Ravanica | IFVCNS, SRB | SRB | Cultivar |
| EUC_TP_391 | Broadway | PGG Wrightson, NZL | NZL | Cultivar |
| EUC_TP_392 | Kontiki | DSV, DEU | DEU | Cultivar |
| EUC_TP_393 | Mercury | ILVO, BEL | BEL | Cultivar |
| EUC_TP_394 | Merian | ILVO, BEL | BEL | Cultivar |
| EUC_TP_395 | Oudenaerdse | ILVO, BEL | BEL | Landrace |
| EUC_TP_396 | Primus | ILVO, BEL | BEL | Landrace |
| EUC_TP_397 | Tp.08.4 | ILVO, BEL | BEL | Breeding material |
| EUC_TP_398 | Tp.08.5 | ILVO, BEL | BEL | Breeding material |
| EUC_TP_399 | Violetta | ILVO, BEL | BEL | Cultivar |
| EUC_TP_400 | Waesse | ILVO, BEL | BEL | Landrace |
| EUC_TP_446 | Affoltern i.E._186 | Agroscope, CHE | CHE | Landrace |
| EUC_TP_447 | Arni b.Biglen_351 | Agroscope, CHE | CHE | Landrace |
| EUC_TP_449 | Horgen_1 | Agroscope, CHE | CHE | Landrace |
| EUC_TP_454 | Lanzenhaeusern_292 | Agroscope, CHE | CHE | Landrace |
| EUC_TP_456 | Riggisberg_311 | Agroscope, CHE | CHE | Landrace |
| EUC_TP_660 | LøRk0498 | Graminor, NOR | NOR | Breeding material |
| EUC_TP_661 | SWA 1575303 | Lantmännen, SWE | SWE | Breeding material |
| EUC_TP_662 | SWA 1576001 | Lantmännen, SWE | SWE | Breeding material |

**Supplementary Table 2**. The best BLASTn-hits in *Arabidopsis thaliana* and *Medicago truncatula* (e-value<1 e-20) of the gene positioned closest to the markers that were significantly associated with freezing tolerance in GWAS analysis . Coverage (%) / identity (%) is given in parentheses.

| **Locus ID** | **Dist.^2^** | **Gene ID** | **Best BLASTn hit in *A. thaliana*** | **Best BLASTn hit in *M. truncatula*** |
| --- | --- | --- | --- | --- |
| LG1_10733810 | 80 | tripr.gene34014 | alpha/beta-Hydrolases superfamily protein (AT3G62860) (57/75) | caffeoylshikimate esterase (LOC11422042), mRNA (74/88) |
| LG1_11608488 | 0 | tripr.gene8902 | inositol transporter 1 (INT1), mRNA (71/70) | inositol transporter 1 (LOC11438245), mRNA (83/92) |
| LG1_20019082 | 0 | tripr.gene36921 | amino acid transporter 1 (AAT1), mRNA (70/66) | cationic amino acid transporter 1 (LOC25482484), mRNA (83/89) |
| LG6_12095124 | 0 | tripr.gene32086 | . | sucrose transport protein SUC8 (LOC11443134), mRNA (18/74) |
| LG3_13453807 | 0 | tripr.gene2834 | beta-hydroxyisobutyryl-CoA hydrolase 1 (CHY1), mRNA (85/71) | 3-hydroxyisobutyryl-CoA hydrolase 1 (LOC25500243), mRNA(100/91) |
| LG2:26124549-26124674/+01 | 0 | tripr.gene2102 | chloride channel C (CLC-C), mRNA (80/73) | chloride channel protein CLC-c (LOC11433847), mRNA (82/91) |
| LG1:4339801-4340029/+24 | 0 | tripr.gene10429 | Peroxidase superfamily protein (PRX52), mRNA (26/70) | cationic peroxidase 1 (LOC11428337), mRNA (92/85) |
|  |  |  |  |  |
| LG3:13143867-13144034/+06 | 0 | tripr.gene1704 | Transducin/WD40 repeat-like superfamily protein (NEDD1), mRNA (44/74) | protein NEDD1 (LOC11411297), mRNA (100/91) |
| scaf_836_20601 | 0 | tripr.gene28991 | ATP/DNA binding protein (AT3G48770), mRNA (36/67) | uncharacterized LOC11421221 (LOC11421221), mRNA (97/90) |
| scaf_153_142327 | 0 | tripr.gene26084 | kinase interacting (KIP1-like) family protein (NET1D), mRNA (8/80) | protein NETWORKED 1A (LOC25485160), transcript variant X2, mRNA (97/90) |

^1^Tp2.0, Legume Information System (legumeinfo.org). ^2^Distance from significant marker (bp).

## Supplementary Figures


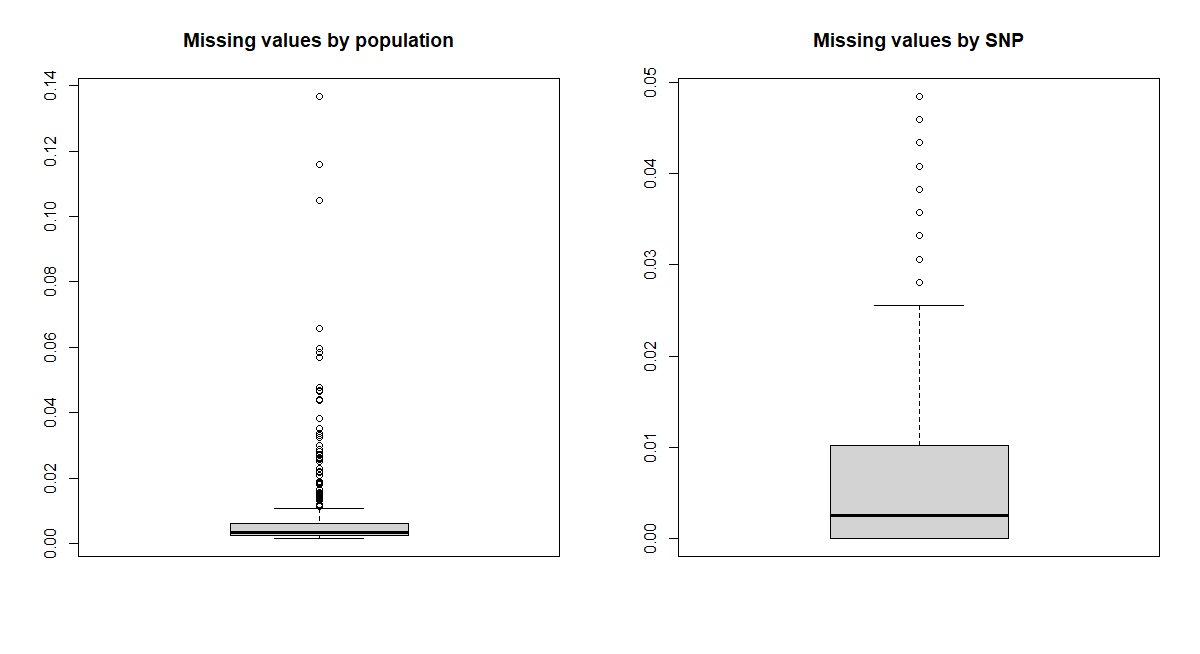


**Supplementary Fig. 1.** Boxplots showing the fraction of missing values by population and SNP, respectively, for the 392 red clover accessions having less than 80% missing values and after removing SNPs with more than 5% missing values and MAF<0.05.


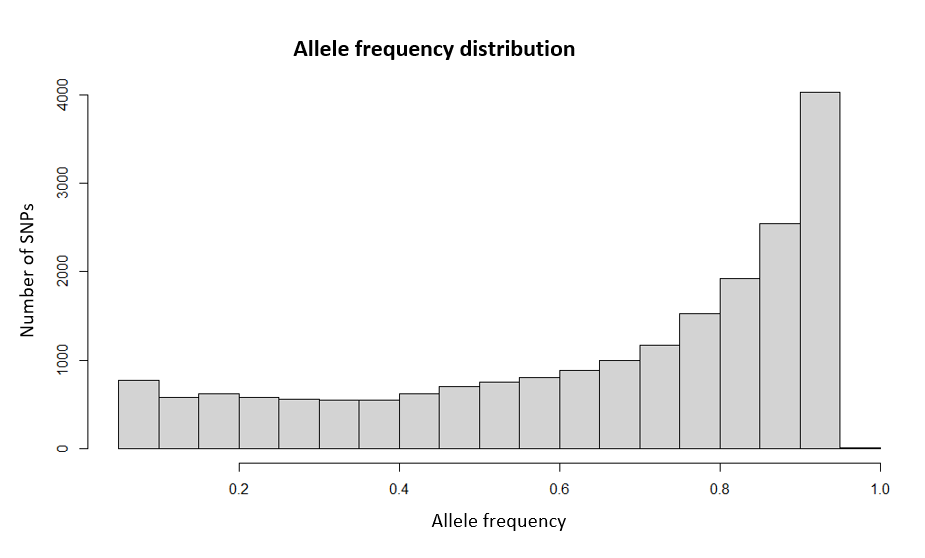


**Supplementary Fig. 2.** Histogram showing the reference allele frequency distribution (across all accessions) after removing SNPs with more than 5% missing values and MAF<0.05.


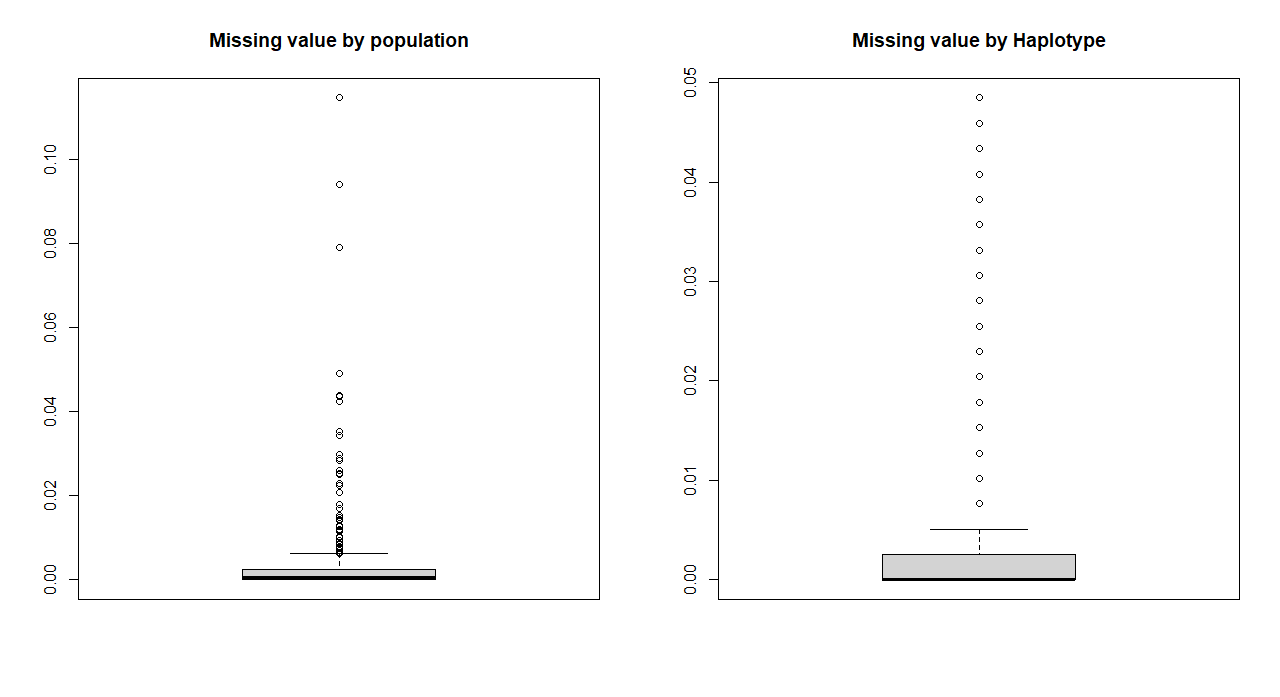


**Supplementary Fig. 3.** Boxplots showing the fraction of missing values by population and haplotype, respectively, for the 392 red clover accessions after removing haplotypes with MAF<0.05 and haplotype polymorphic loci (HTPs) with more than 5% missing values.


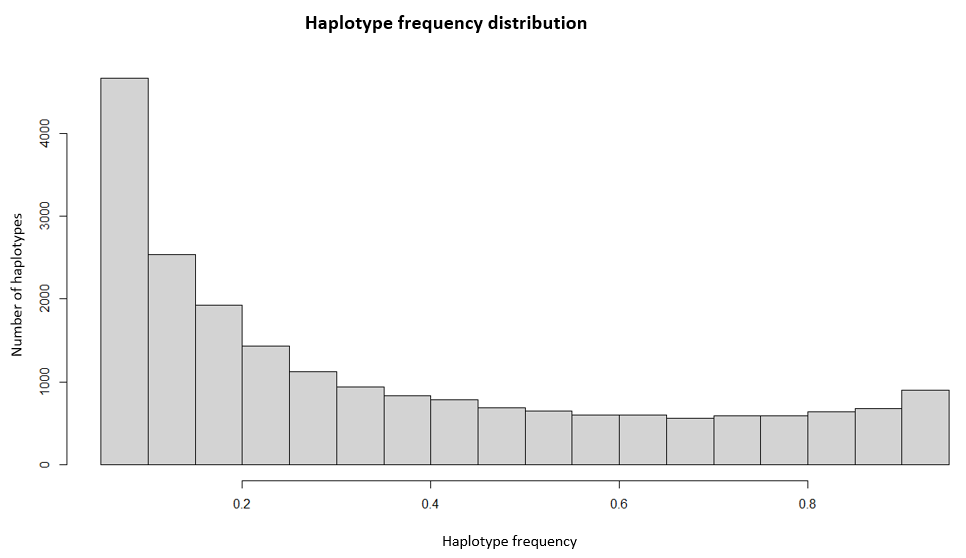


**Supplementary Fig. 4.** Histogram showing the haplotype frequency distribution (across all accessions) after removing haplotypes with MAF<0.05 and haplotype polymorphic loci (HTPs) with more than 5% missing values.

**A**


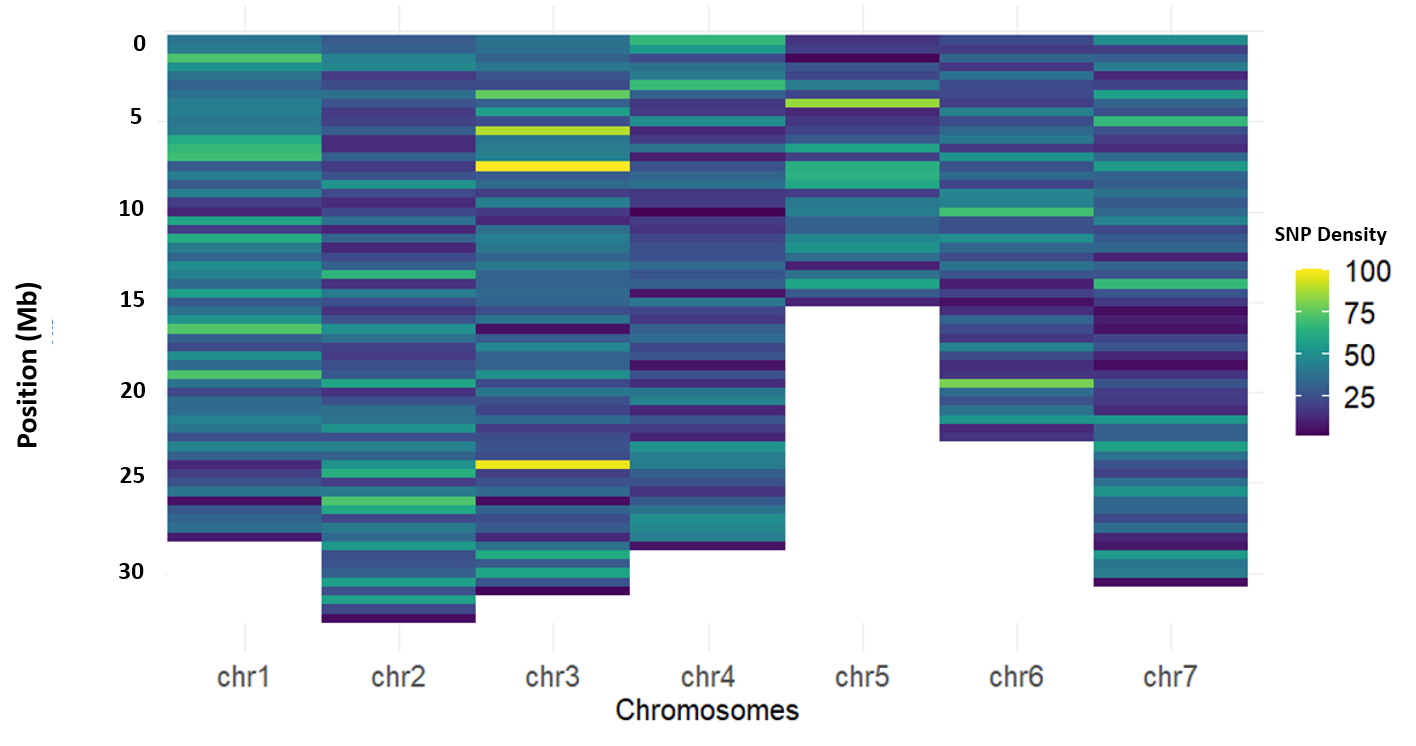


**B**


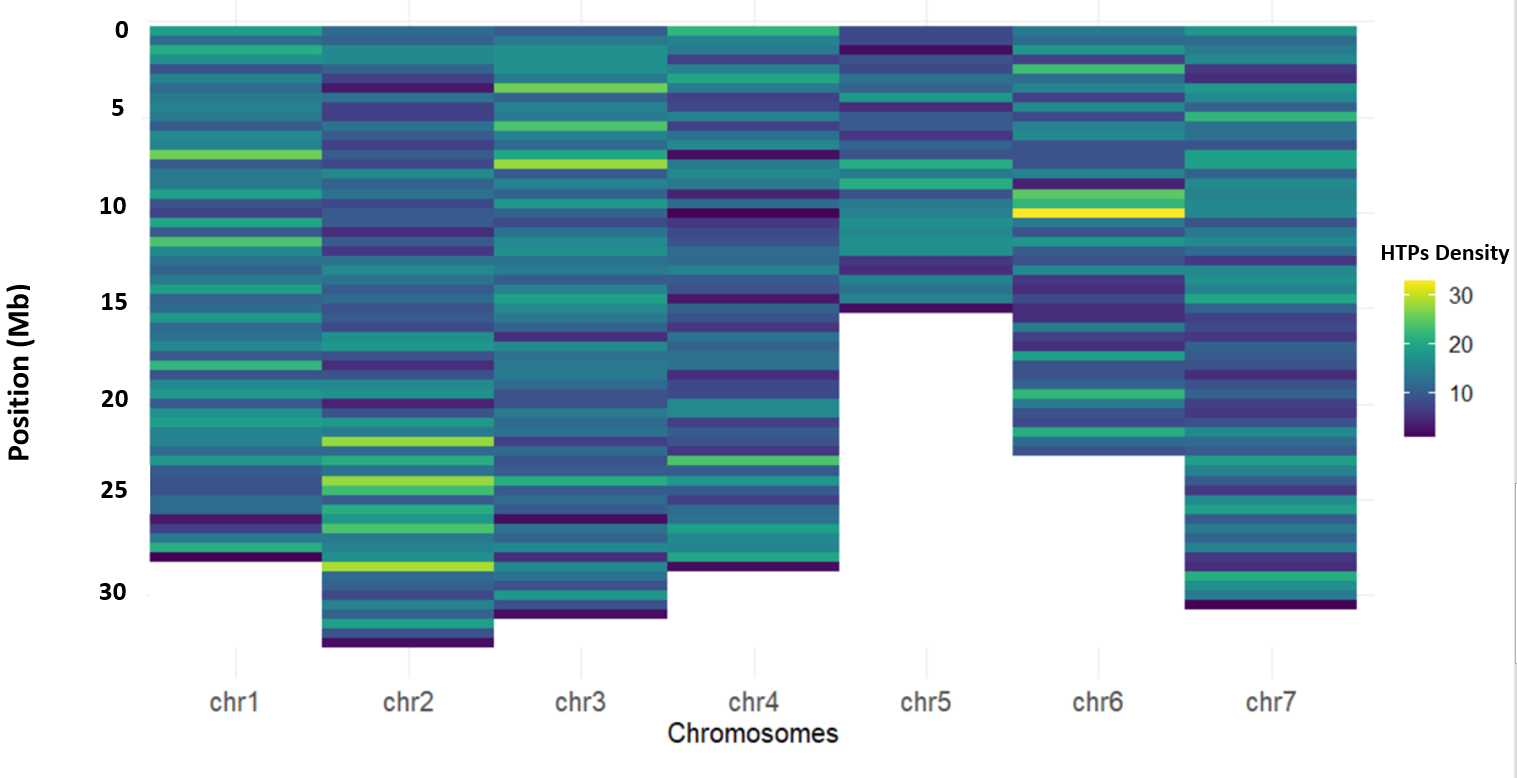


**Supplementary Fig. 5.** Heat maps of each chromosome representing of SNPs (**A**) and HTPs (**B**) density within windows of 500 kb. The y-axis reports the distance in Mbp.


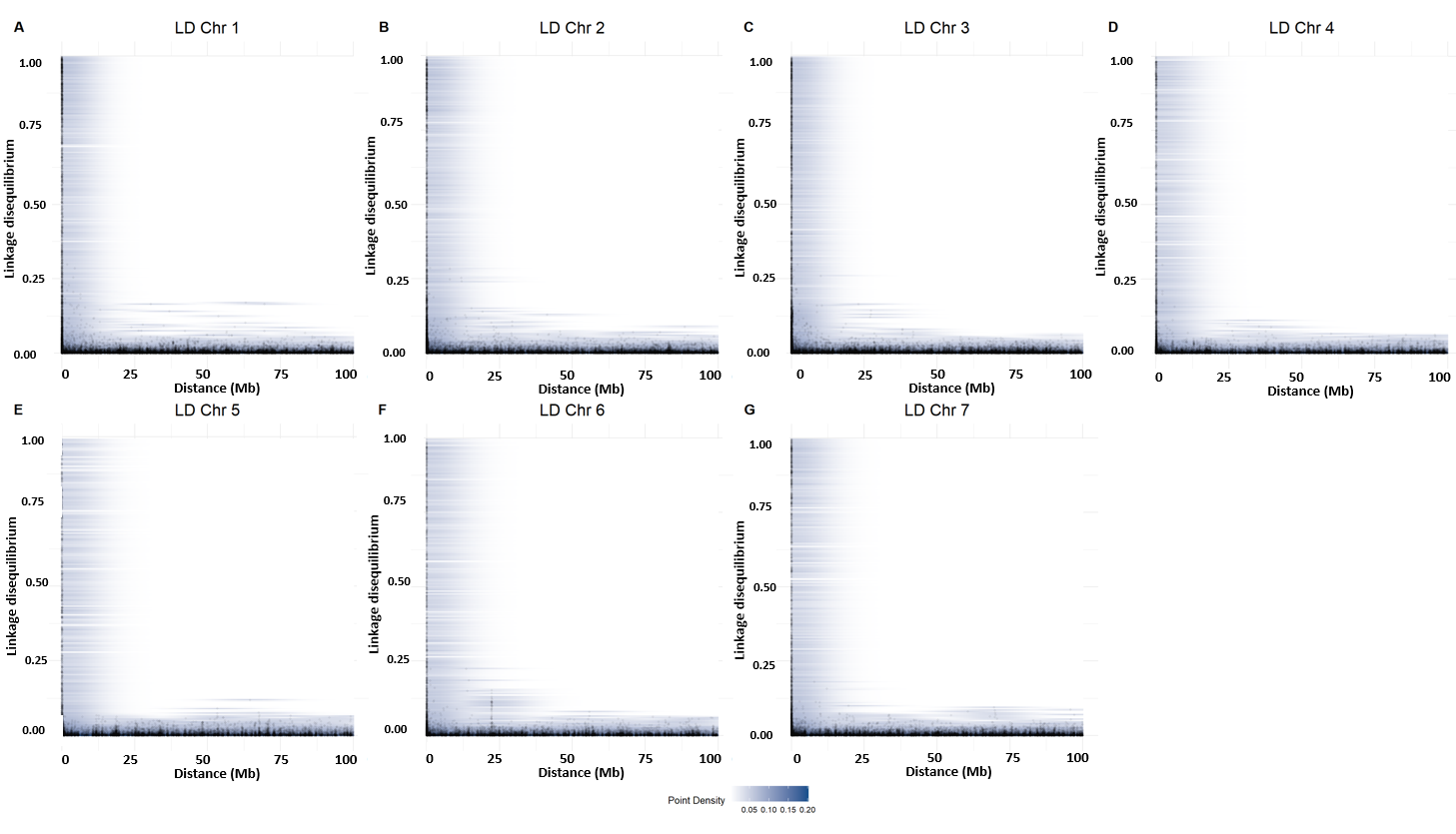
**Supplementary Fig. 6.** Linkage disequilibrium (LD) decay in the seven chromosomes (**A-G**) as scatter plot of squared partial correlations between reference allele frequencies of pairs of SNP markers and genomic distance between SNP markers in Mb.


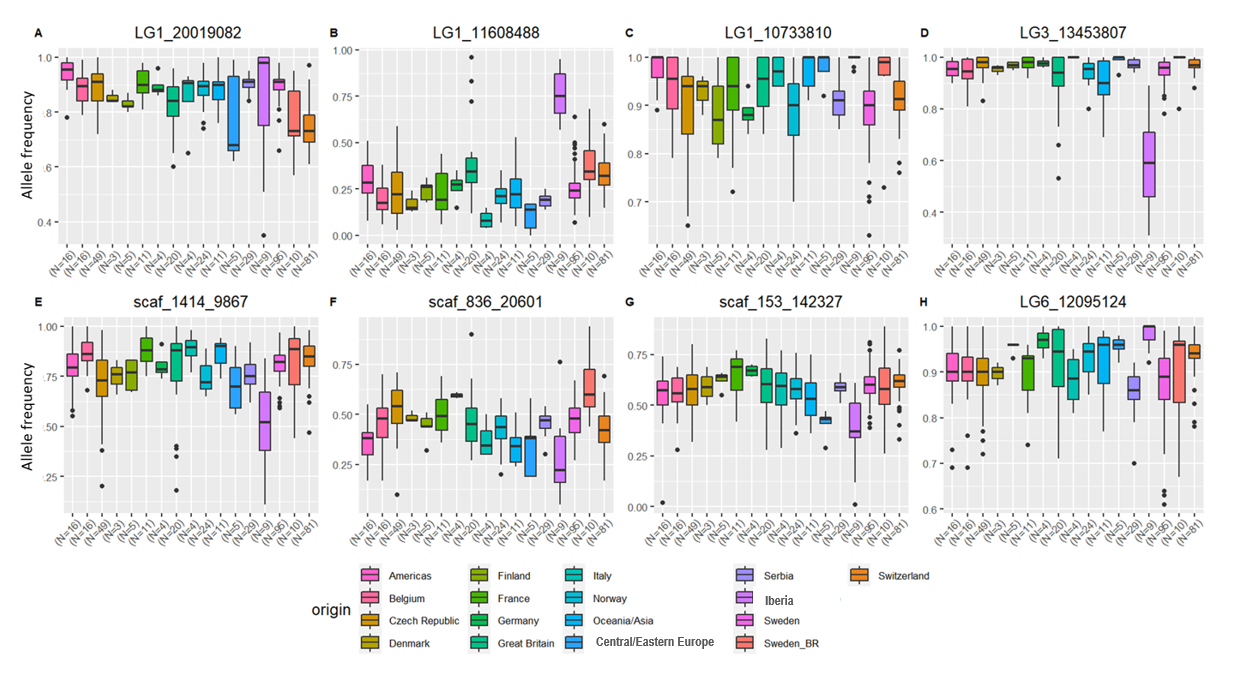


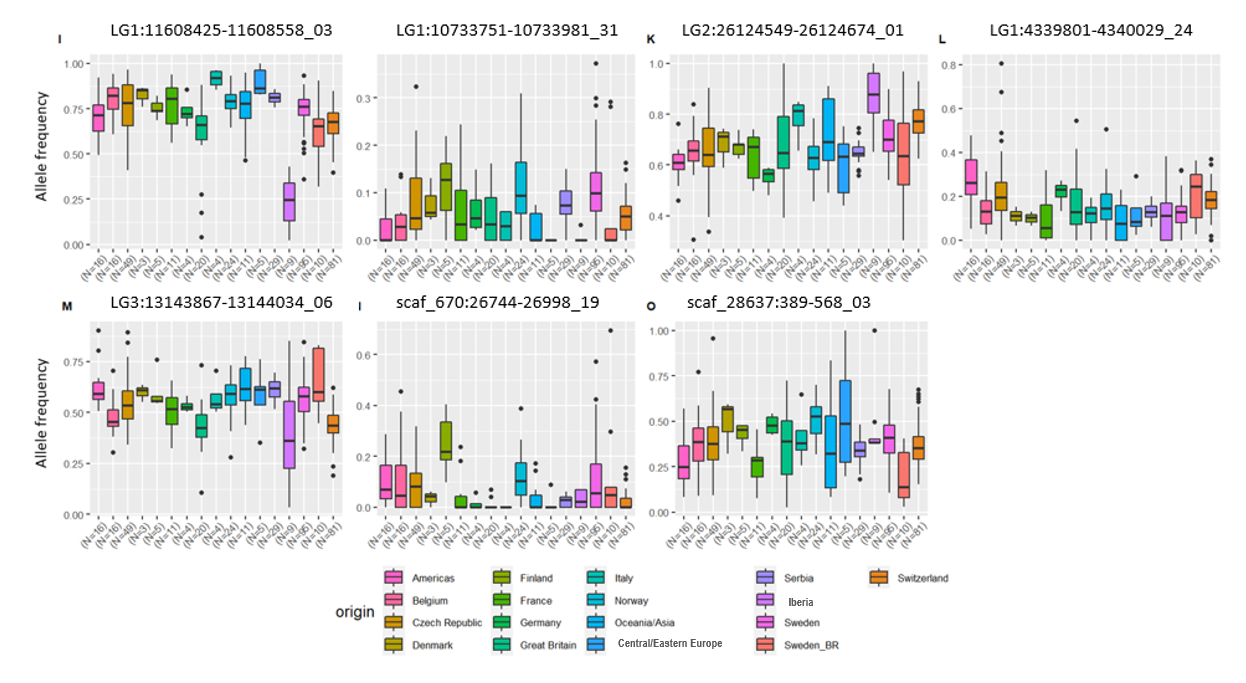


**Supplementary Fig. 7.** Boxplots showing the variation/distribution of allele frequencies for the SNPs (**A**-**H**) and haplotypes (**I**-**O**) significantly associated with FT for red clover accessions grouped by country/area of origin. The Breeding material of Sweden (Sweden_BR) was kept as separate group.


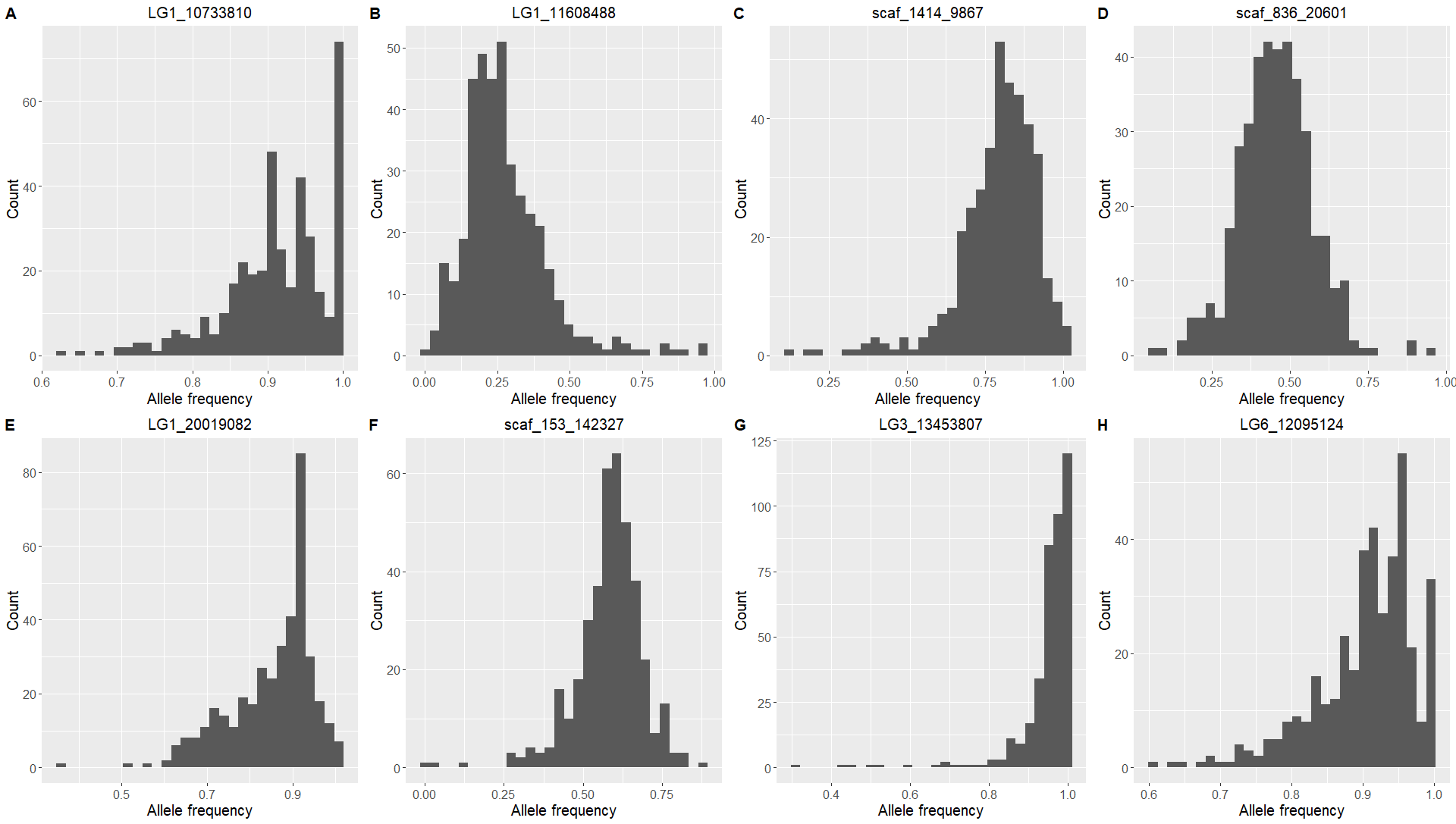


**Supplementary Fig. 8.**Reference allele frequency distribution of SNP markers significantly associated with freezing tolerance in a GWAS across 392 red clover accessions.


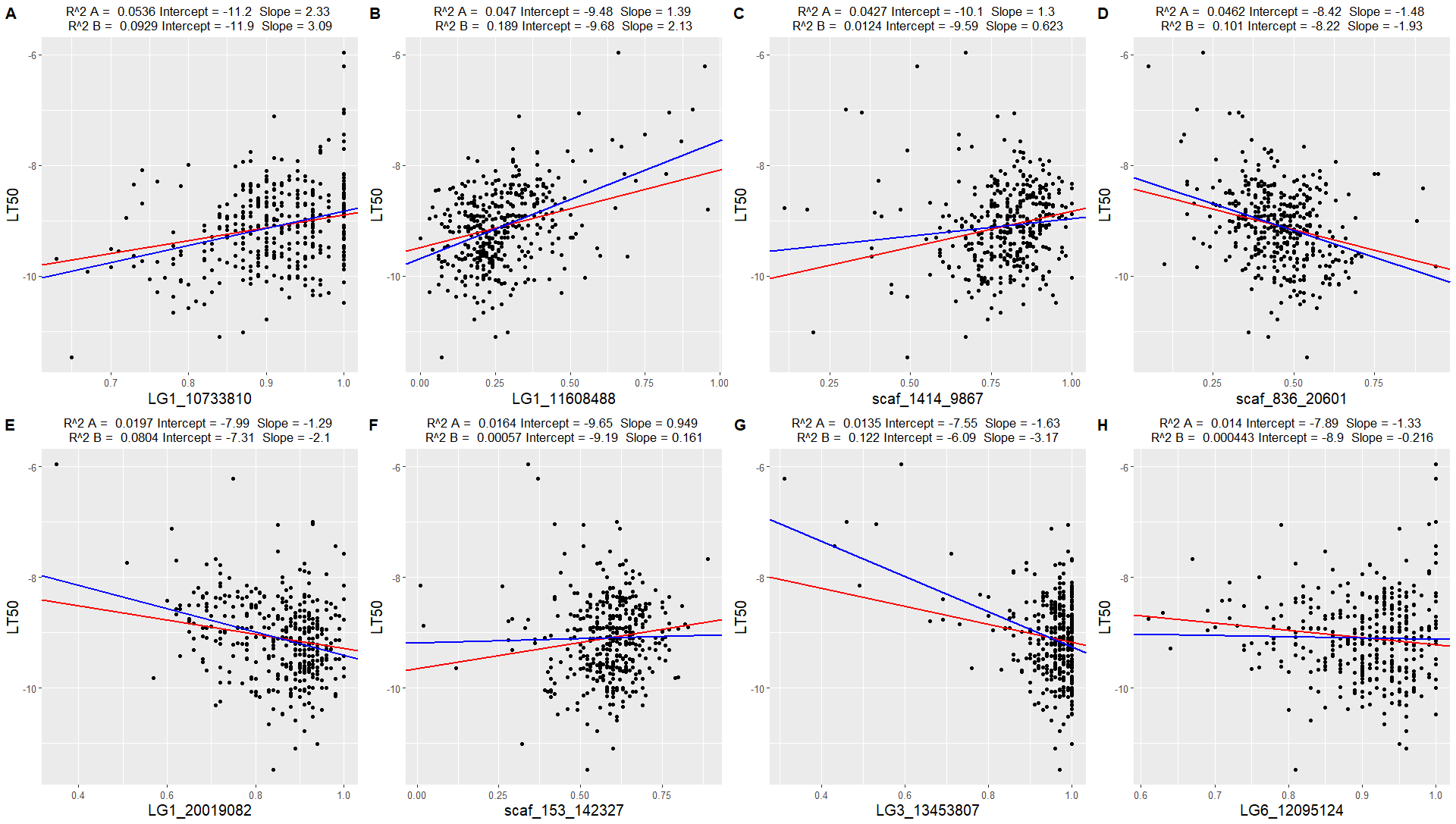


**Supplementary Fig. 9.**Regression lines for the reference allele frequency against freezing tolerance (LT50) for SNP markers significantly associated with freezing tolerance in a GWAS across 392 red clover accessions. GWAS models accounting for kinship (red lines) and GWAS models not accounting for kinship (blue lines) are plotted. $R^{2}$and slope values are given in each plot for both models with and without kinship (“$R^{2}$A” and “$R^{2}$B” at the top of each regression plots, respectively).


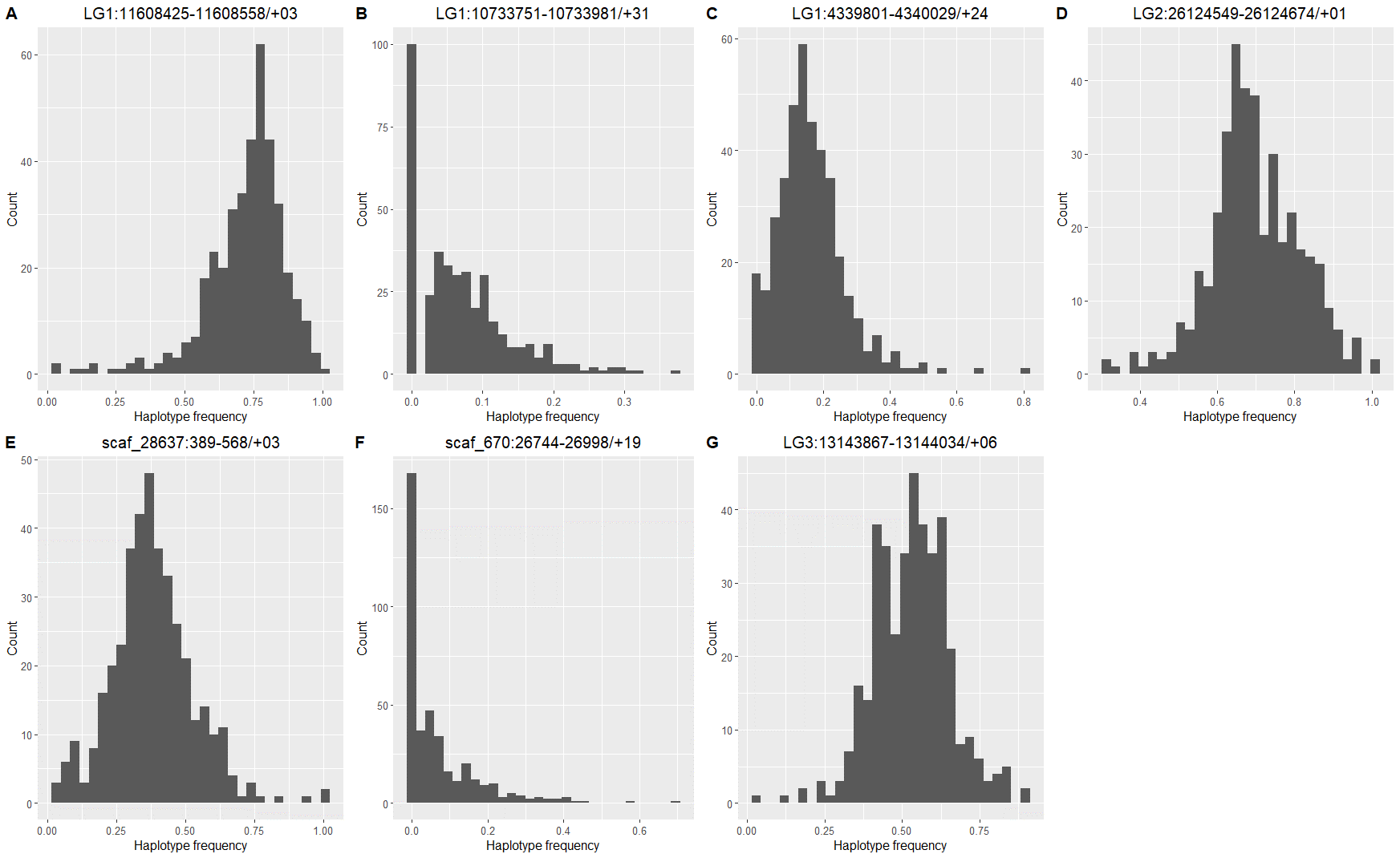


**Supplementary Fig. 10.**Reference allele frequency distribution of haplotype markers significantly associated with freezing tolerance in a GWAS across 392 red clover accessions.


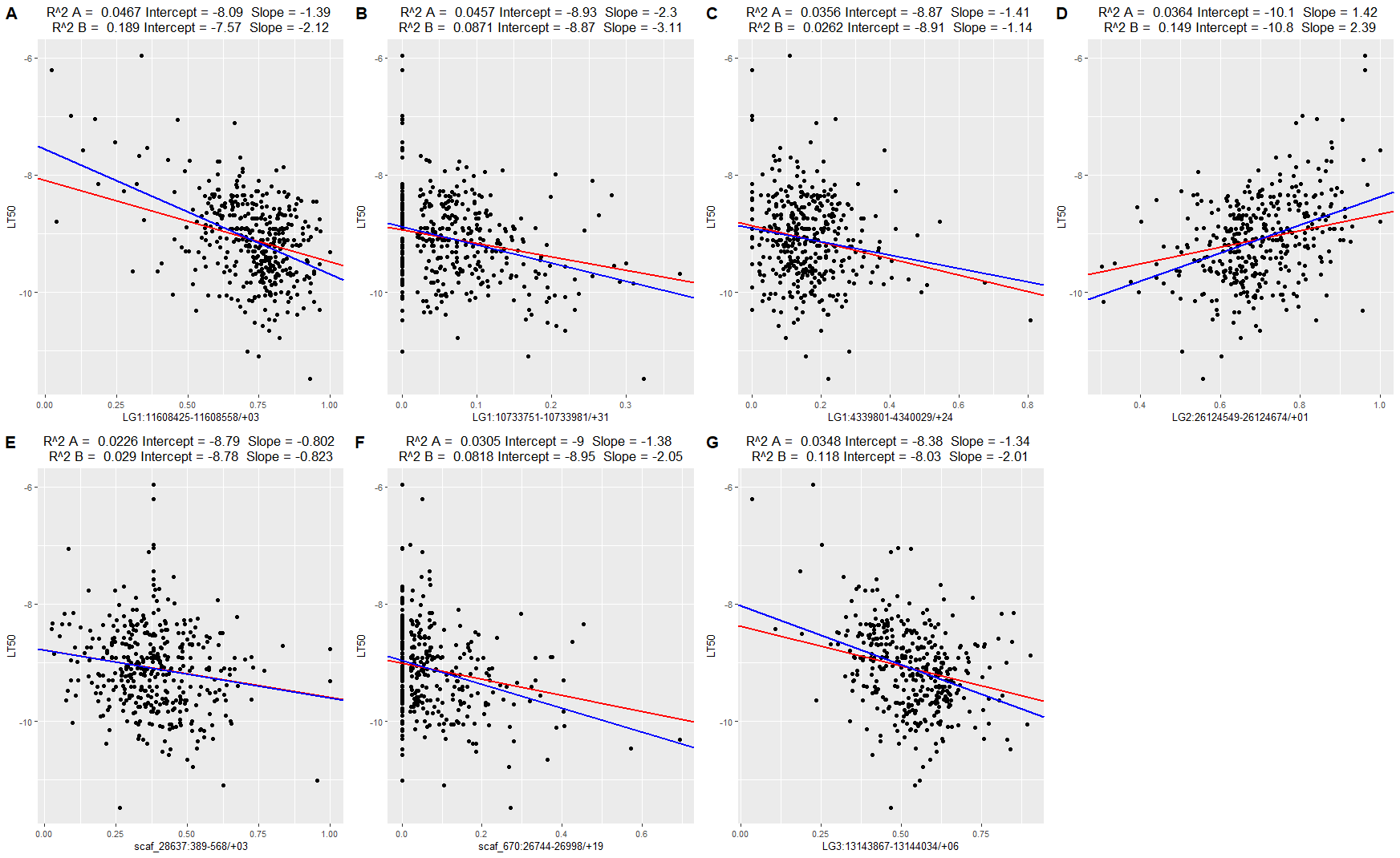


**Supplementary Fig. 10.**Regression lines for the reference allele frequency against freezing tolerance (LT50) for haplotype markers significantly associated with freezing tolerance in a GWAS across 392 red clover accessions. GWAS models accounting for kinship (red lines) and GWAS models not accounting for kinship (blue lines) are plotted. $R^{2}$and slope values are given in each plot for both models with and without kinship (“$R^{2}$A” and “$R^{2}$B” at the top of each regression plots, respectively).
